# Rhizosphere-derived *Stutzerimonas stutzeri* AUMC B-503: a promising biocontrol and plant growth-promoting strain for managing brown spot disease in rice (*Oryza sativa*)

**DOI:** 10.3389/fpls.2025.1700440

**Published:** 2025-12-11

**Authors:** Sally M. Metwally, Shimaa El-Sapagh, Sameh S. Ali, Jianzhong Sun, Michael Schagerl, Doaa E. Elsherif

**Affiliations:** 1 Botany and Microbiology Department, Faculty of Science, Tanta University, Tanta, Egypt; 2 Biofuels Institute School of the Environment Safety Engineering, Jiangsu University, Zhenjiang, China; 3 Department of Functional and Evolutionary Ecology, University of Vienna, Vienna, Austria

**Keywords:** plant growth-promoting bacteria (PGPB), *Stutzerimonas stutzeri*, *Oryza sativa*, *Bipolaris oryzae*, *Phragmites australis*, biocontrol, sustainable agriculture

## Abstract

Brown spot disease caused by the fungus *Bipolaris oryzae* severely limits rice production and quality worldwide. The excessive use of chemical fungicides underscores the need for sustainable biological alternatives, such as plant growth-promoting rhizobacteria (PGPR). In this study, four Gram-negative bacterial isolates, designated as P1–P4, were obtained from the rhizosphere of *Phragmites australis* and evaluated for their plant growth-promoting and antifungal activities. Among these isolates, P3 (molecularly identified as *Stutzerimonas stutzeri* AUMC B-503) exhibited the highest production of indole-3-acetic acid (IAA) and hydrogen cyanide (HCN), along with strong phosphate-solubilizing capacity and robust biofilm formation. Dual-culture assays revealed that this strain significantly inhibited the mycelial growth of *B. oryzae*, indicating potent antifungal activity. In agarose-based and pot experiments, AUMC B-503 significantly increased the shoot and root length, biomass, total soluble carbohydrates, and photosynthetic pigment contents of rice seedlings compared with untreated controls. Moreover, inoculated plants exhibited reduced levels of malondialdehyde (MDA) and hydrogen peroxide (H_2_O_2_), accompanied by enhanced activities of antioxidant enzymes [polyphenol oxidase (PPO), peroxidase (POD), phenylalanine ammonia-lyase (PAL), and ascorbate peroxidase (APX)] and higher levels of phenolics, flavonoids, ascorbic acid, and total antioxidant capacity. At the transcriptional level, bacterial treatment upregulated the gene expression of *OsCHS*, *OsCHI*, and *OsFLS*, corresponding to the observed increase in total flavonoids. Additionally, the expression of the *OsOAT* and *OsERF83* genes was also elevated, suggesting improved proline metabolism and ethylene/jasmonate-mediated stress signaling. These integrated physiological, biochemical, and molecular responses demonstrate that AUMC B-503 promotes rice growth and enhances tolerance to *B. oryzae* infection by mobilizing nutrients, activating antioxidants, and inducing the transcription of defense-related pathways. The results highlight AUMC B-503 as a promising and low-risk candidate for promoting rice growth and suppressing brown spot under controlled conditions. The study provides mechanistic evidence for its efficacy while recognizing that biosafety, non-target, and regulatory evaluations are prerequisites for field application.

## 1 Introduction

Rice (*Oryza sativa* L.) is a fundamental staple food for more than half of the global population and remains the third most extensively produced cereal after maize and wheat (Fukagawa and Ziska, 2019; Hashim et al., 2024). Beyond its dietary role, rice sustains the livelihoods of millions of smallholder farmers and underpins food security across developing regions. In Egypt, it holds considerable economic importance as the second most crucial export crop after cotton, contributing significantly to the national economy and rural household income (Shabana et al., 2008). As the global population is projected to surpass nine billion by 2050, rice production must increase by at least 30% before 2030 to meet the escalating food demand (Bin Rahman and Zhang, 2023). However, achieving this goal is increasingly challenged by the combined pressures of climate change and pathogen outbreaks, which exacerbate abiotic and biotic stresses, thereby drastically reducing yield stability (Hashim et al., 2024).

Among biotic stresses, brown spot disease, caused by the fungus *Bipolaris oryzae*, is one of the most prevalent and destructive infections affecting rice cultivation globally. The pathogen infects leaves, glumes, and spikelets throughout plant development, resulting in necrotic lesions and substantial yield and quality losses ranging from 6% to 90%, depending on the severity of infection and environmental conditions (Ashfaq et al., 2021; Keerthana et al., 2022). In Egypt, brown spot is recognized as the second most significant rice disease after blast, leading to measurable decreases in grain quality and harvestable yield (Bekheet et al., 2021). Chemical fungicides remain the primary means of controlling brown spot and other rice diseases; however, their sustainability is declining. Excessive and prolonged fungicide use has escalated production costs and contributed to soil and water contamination, phytotoxicity, and the emergence of fungicide-resistant *B. oryzae* strains. Additionally, agrochemical residues pose health risks to both farmers and consumers. These limitations highlight an urgent need for eco-friendly and biologically based disease management strategies that align with sustainable agricultural goals (Khoso et al., 2024).

Plant growth-promoting rhizobacteria (PGPR), particularly those belonging to the genus *Pseudomonas*, have garnered attention as biocontrol agents and biofertilizers due to their multifaceted mechanisms of action, encompassing both direct and indirect effects on plant growth and disease suppression. They synthesize antifungal metabolites, siderophores, hydrogen cyanide (HCN), and hydrolytic enzymes that inhibit phytopathogens while simultaneously promoting plant health through phosphate solubilization, nitrogen fixation, and the production of growth-regulating phytohormones such as indole-3-acetic acid (IAA) (Khalil et al., 2015; Singh et al., 2022; Khoso et al., 2024). Recently, the genus *Pseudomonas* was split into several genera based on phylogenetic studies (Lalucat et al., 2022). Among the new recombined species, *Stutzerimonas stutzeri* (formerly *Pseudomonas stutzeri*) stands out for its exceptional metabolic adaptability, stress tolerance, and effective root-colonizing ability, positioning it as a promising candidate for agrobiotechnological applications (Gao et al., 2024). Nevertheless, the potential of *S. stutzeri* in suppressing rice diseases and its ecological diversity in association with non-crop plant hosts remains insufficiently explored.

The rhizosphere of medicinal and wetland plants, such as *Phragmites australis* (common reed), represents a biologically rich yet underexplored habitat that harbors diverse bacterial communities adapted to chemically complex environments. *P. australis* has a cosmopolitan distribution in wetlands and is highly competitive. It exhibits well-documented antimicrobial, antioxidant, and allelopathic activities, reflecting the bioactive composition of its root exudates (Shaheen et al., 2019; Kalu et al., 2022). Such conditions favor the selection of metabolically versatile microorganisms with inherent defensive and growth-promoting properties. Investigating the microbiota associated with this plant, therefore, provides an opportunity to identify beneficial microbial strains that can enhance crop resilience and support sustainable, low-input agricultural practices aligned with the United Nations’ Sustainable Development Goals (SDGs) (United Nations, 2024).

The present study aimed to isolate and characterize *Stutzerimonas* strains with potential biocontrol activity from the rhizosphere of *P. australis* and to evaluate their potential as plant growth-promoting and biocontrol agents against *B. oryzae*, the causal pathogen of rice brown spot disease. Specifically, this work aimed to identify a highly effective strain based on its biochemical and physiological traits, assess its ability to enhance growth and antioxidant defenses in rice under both normal and pathogen-challenged conditions, and elucidate its possible mechanisms through physiological, biochemical, and molecular analyses.

## 2 Materials and methods

### 2.1 Experimental plant material

Seeds of rice (*O. sativa* L., cv. ‘Giza 179’) were obtained from the Rice Research and Training Center (RRTC), Sakha, Kafr El-Sheikh, Egypt, and used in all experiments.

### 2.2 Soil sampling and isolation of rhizobacterial strains

Soil samples were collected from the rhizosphere of *P. australis* plants growing naturally along irrigation channels in Tanta, El-Gharbia Governorate, Egypt (30.7862°N, 31.0004°E). Sampling was conducted during the summer season (June 2023–July 2024). The sampling site represents a typical Nile Delta wetland ecosystem characterized by clay-loam soil with moderate salinity and high organic content. Intact *P. australis* plants were carefully uprooted with approximately 15 cm of surrounding rhizospheric soil to preserve root–soil integrity. Soil tightly adhering to the roots was aseptically transferred into sterile polyethylene bags and transported to the laboratory under cooled conditions for immediate processing following standard rhizosphere sampling procedures (Barillot et al., 2013).

Serial dilutions (10^−1^–10^−6^) of rhizospheric soil suspensions were prepared in sterile saline solution and plated on King’s B agar (KBA), nutrient agar (NA), and tryptic soy agar (TSA) (van der Linde et al., 1999; Mazumdar et al., 2007; Devika et al., 2021). These media are suitable for isolating fluorescent *Pseudomonas* (*Stutzerimonas*) species. Plates were incubated at 28 °C ± 2°C for 24–48 h. Colonies with distinct morphologies were then selected and re-streaked on KBA to obtain pure cultures. Isolates were differentiated based on colony pigmentation (fluorescent *vs*. non-fluorescent), shape, margin, and texture, following the diagnostic criteria described by Cottyn et al. (2009). Four morphologically distinct isolates (P1–P4) were purified and maintained on KBA slants at 4°C for short-term storage and in 20% (v/v) glycerol at −20°C for long-term preservation. Negative control plates (uninoculated media) were incubated alongside experimental plates to verify aseptic conditions. Preliminary characterization of isolates included Gram staining (Collee et al., 1996) and catalase and oxidase tests (Taylor and Achanzar, 1972; Al-Joda and Jasim, 2021). The ultrastructural morphology of representative isolates was examined using scanning electron microscopy (SEM) with a JSM-6510 LV (JEOL, Tokyo, Japan) at an accelerating voltage of 30 kV, following the procedure described by Hartmann et al. (2010), at the Electron Microscopy Unit, Faculty of Agriculture, Mansoura University, Egypt.

### 2.3 Biosafety evaluation for the most potent strain

The most potent bacterial isolate was evaluated for basic biosafety characteristics, including coagulase activity (Salehi, 2022), hemolytic activity (Slots, 1992), and antibiotic susceptibility determined using the Kirby–Bauer disk diffusion method (Karatuna and Yagci, 2010). The antibiotic resistance profile was assessed against clinically important antibiotic classes, including β-lactam cephalosporins, quinolones, macrolides, monobactams, and aminoglycosides. Antibiotic discs were obtained from Oxoid Ltd. (Basingstoke, UK) and used at standard concentrations (**Supplementary Table 1**). Following incubation at 37°C for 18 h under aerobic conditions, zones of growth inhibition were measured and interpreted according to European Committee on Antimicrobial Susceptibility Testing (EUCAST) clinical breakpoints (Hombach et al., 2012).

### 2.4 Collection and isolation of *B. oryzae*

The fungal pathogen *B. oryzae*, the causal agent of rice brown spot disease, was isolated from naturally infected leaves of *O. sativa* plants exhibiting characteristic necrotic lesions. Symptomatic samples were collected from a rice field in Kafr El-Sheikh, Egypt, and immediately placed in sterile Ziploc bags for transport to the laboratory under cooled conditions (4°C). Infected leaf segments (approximately 5 mm^2^) were surface-sterilized in 1% (v/v) sodium hypochlorite solution for 1 min, followed by three successive rinses with sterile distilled water to remove any residual disinfectant. The sterilized tissues were aseptically blotted dry on sterile filter paper and placed on potato dextrose agar (PDA) plates. Cultures were incubated at 28 °C ± 2°C for 3 days. Negative controls (uninoculated media) were incubated alongside experimental plates to confirm the absence of contamination during culture and incubation. Emerging fungal colonies were sub-cultured to obtain pure isolates and maintained on PDA slants at 4°C for subsequent pathogenicity and molecular identification assays (Karan et al., 2021; Ejigu et al., 2024). *B. oryzae* was examined microscopically for morphological characterization. Colony growth pattern, mycelial color, sporulation intensity, conidial shape, and pigmentation were observed and documented. Microscopic observations were conducted and photographed using an Olympus AX70 microscope.

### 2.5 Molecular and phylogenetic identification

Genomic DNA of the selected fungal (*B. oryzae*) and bacterial P3 isolates was extracted to confirm their molecular identities and phylogenetic placements. Fungal DNA extraction was performed at the Assiut University Mycological Centre (AUMC), Assiut University, Egypt, using the Solg™ Genomic DNA Prep Kit (SolGent, Daejeon, South Korea). The internal transcribed spacer (ITS) region of the ribosomal DNA was amplified with the universal primers ITS1 (5′-TCCGTAGGTGAACCTGCGG-3′) and ITS4 (5′-TCCTCCGCTTATTGATATGC-3′) following White et al. (1990). For bacterial identification, the bacterial isolate was cultivated in nutrient broth at 28°C for 48 h, and genomic DNA was extracted using the Patho Gene-spin DNA/RNA Extraction Kit (Intron Biotechnology, Seongnam, South Korea) according to El-Sapagh et al. (2023). The 16S rRNA gene was amplified using the universal primers 27F (5′-AGAGTTTGATCCTGGCTCAG-3′) and 1492R (5′-GGTTACCTTGTTACGACTT-3′). PCR products from both isolates were purified with the SolGent PCR Purification Kit-Ultra and subjected to bidirectional sequencing. Two sequence datasets were analyzed: the fungal dataset (GenBank accession PP946180.1) comprising 20 taxa and 793 aligned nucleotide sites, and the bacterial dataset (OQ672786.1) comprising 31 taxa and 1,556 aligned sites. Multiple sequence alignments and phylogenetic analyses were conducted using MEGA version 12.0.15 (Kumar et al., 2024). Phylogenetic trees were inferred using the neighbor-joining (NJ) method (Saitou and Nei, 1987) based on evolutionary distances computed using the Maximum Composite Likelihood (MCL) model (Tamura et al., 2004). The analysis included all codon positions and non-coding regions. Statistical support for clades was assessed using 1,000 bootstrap replicates (Felsenstein, 1985), with values of 70% or higher being considered significant. Gaps and missing data were treated using the pairwise deletion option to maximize information content across sequences of varying lengths. Phylogenetic computations assumed uniform substitution rates and homogeneous evolutionary patterns among lineages. All analyses were performed in the MEGA 12 graphical interface (Windows platform) with parallel processing enabled (three threads) to optimize performance. The fungal sequence was deposited in GenBank under accession number PP946180 (*B. oryzae* AUMC 16423), whereas the bacterial 16S rRNA sequence was deposited under accession number OQ672786 (*S. stutzeri* AUMC B-503).

### 2.6 Assessment of biocontrol and plant growth-promoting traits of bacterial isolates

The four bacterial isolates were evaluated for key biochemical traits associated with promoting plant growth and suppressing disease. These included the production of HCN, phosphate solubilization, IAA production, and biofilm formation—traits known to enhance rhizosphere competence and antagonistic potential toward phytopathogens.

#### 2.6.1 HCN production

HCN production was assessed using the picrate–carbonate paper assay on KBA, adapted for semi-quantitative spectrophotometric measurement at 625 nm (Reetha et al., 2014). Briefly, each isolate was streaked on KBA (HiMedia, Mumbai, India) supplemented with glycine (4.4 g·L^−1^) to enhance cyanogenesis. A sterile Whatman No. 1 filter paper strip impregnated with picric acid (0.5%, w/v) and Na_2_CO_3_ (2%, w/v) was affixed to the lid of each Petri dish. Plates were sealed with parafilm and incubated at 28 °C ± 2°C for 3–4 days. Formation of a reddish-brown isopurpurate on the indicator paper signified HCN release. The papers were then eluted in 10 mL of distilled water, and absorbance was recorded using a UV–VIS spectrophotometer (Shimadzu UV-1800, Kyoto, Japan) at 625 nm against a reagent-only blank; uninoculated KBA plates were included as negative controls. Each assay was performed in triplicate, and results were reported as OD_625_ values, which provided a relative index of cyanide production under identical assay conditions, as previously described for rhizobacteria.

#### 2.6.2 Phosphate solubilization

The phosphate-solubilizing capacity of bacterial isolates was determined according to Nautiyal (1999). A 10-µL aliquot of each bacterial suspension (~10^8^ CFU·mL^−1^) was spot-inoculated onto Pikovskaya’s agar medium (HiMedia, India) containing 0.5% (w/v) tricalcium phosphate [Ca_3_(PO_4_)_2_] as an insoluble phosphate source. Plates were incubated at 28 °C ± 2°C for 7 days, and phosphate solubilization was evidenced by the formation of a clear halo surrounding the colony. The solubilization index was calculated as the ratio of total diameter (colony + halo) to colony diameter. All assays were conducted in triplicate, and results were expressed as mean ± standard error.

#### 2.6.3 IAA production

Quantitative estimation of IAA production by bacterial isolates was performed using the colorimetric method described by Das et al. (2019). Bacterial cultures were grown in 20 mL of sucrose–minimal salt (SMS) broth [per liter: 10 g sucrose, 2.0 g K_2_HPO_4_, 1.0 g (NH_4_)_2_SO_4_, 0.5 g MgSO_4_·7H_2_O, 0.5 g CaCO_3_, 0.1 g NaCl, and 0.5 g yeast extract; pH 7.2] supplemented with 0.5 mg·mL^−1^l-tryptophan as a precursor. Cultures were incubated for 5 days at 28°C ± 2°C with shaking at 150 rpm. Uninoculated SMS broth served as a negative control. After incubation, 1 mL of culture supernatant was mixed with 2 mL of Salkowski’s reagent (prepared by dissolving 1 mL of 0.5 M FeCl_3_ in 50 mL of 35% HClO_4_ solution) and incubated at room temperature for 20 min in the dark to allow the development of a pink color. Absorbance was recorded at 530 nm using a UV–VIS spectrophotometer (Shimadzu UV-1800, Japan). The IAA concentration was calculated using a standard calibration curve prepared with pure IAA (10–50 mg·L^−1^; R^2^ ≥ 0.99). All assays were performed in triplicate, and results were expressed as mean ± standard error.

#### 2.6.4 Biofilm formation

The biofilm-forming ability of the tested bacterial isolates was evaluated using the microtiter plate (MTP) assay described by Stepanović et al. (2007), with modifications according to Coffey and Anderson (2014). Each isolate was first cultured in 5 mL of tryptic soy broth (TSB) supplemented with 1% (w/v) glucose and incubated at 37°C for 24 h. The cultures were then diluted 1:10 with fresh TSB–glucose medium. Aliquots (200 µL) of the diluted bacterial suspension were dispensed into sterile 96-well flat-bottom microtiter plates (Corning, Corning, New York, USA). Wells containing sterile, uninoculated broth served as negative controls. Plates were incubated without agitation at 37°C for 24 h to allow biofilm formation. Non-adherent cells were gently removed by rinsing the wells three times with sterile phosphate-buffered saline (PBS; pH 7.2). The adherent biofilms were fixed with 2% sodium acetate for 15 min and stained with 0.1% (w/v) crystal violet for 20 min. Excess stain was removed by washing with distilled water, and the bound dye was solubilized with 33% (v/v) glacial acetic acid. Biofilm biomass was quantified by measuring the optical density at 570 nm using a microplate reader (Model EMR-500, Labomed, Los Angeles, California, USA). All assays were performed in triplicate. Based on OD_570_ values, isolates were classified as non-former or weak, moderate, or strong biofilm formers according to Stepanović et al. (2007).

### 2.7 Antifungal activity of bacterial isolates against *B. oryzae*

The antifungal potential of the four bacterial isolates was evaluated against *B. oryzae* using a modified dual-culture plate assay described by Chandra et al. (2020). Petri dishes (9 cm in diameter) containing PDA were marked with four equidistant lines, each positioned 1 cm from the plate margins and arranged perpendicularly to one another. Bacterial isolates (P1, P2, P3, and P4) were streaked along these lines, while an 8-mm mycelial disc from a 7-day-old *B. oryzae* culture was aseptically placed at the center of each plate. Plates were incubated at 28 °C ± 2°C for 8 days. Control plates containing only the fungal disc (without bacterial inoculation) were included to determine normal fungal growth. The antifungal activity was quantified by calculating the percentage inhibition of radial mycelial growth using the following formula:

Inhibition (%) = [(*R*_1_ − *R*_2_)/*R*_1_] × 100,

where *R*_1_ is the radial growth of the pathogen in the control plate and *R*_2_ is the radial growth in the presence of the bacterial isolate. All experiments were performed in triplicate, and results were expressed as mean ± standard error.

### 2.8 Experimental design for seedling growth promotion of *O. sativa* L. using an agarose-based assay

The plant growth-promoting potential of the bacterial isolates (P1, P2, P3, and P4) was evaluated using an agarose-based bioassay, following the general approach of Verma et al. (2018), with modifications to enhance reproducibility. Seeds of *O. sativa* L. (cv. Giza-179) were surface-sterilized by immersion in 4% (v/v) sodium hypochlorite solution under constant agitation for 20 min, followed by rinsing five times with sterile distilled water. Subsequently, the seeds were immersed in 95% ethanol for 3–5 min, rinsed thoroughly again with sterile distilled water, and air-dried under aseptic conditions. The bacterial inocula were prepared from 24-h cultures of the most bioactive isolates grown in nutrient broth and adjusted to a final concentration of approximately 1 × 10^7^ CFU·mL^−1^ using sterile saline (0.85%, w/v). Fifty sterilized rice seeds were soaked in 5 mL of each bacterial suspension for 48 h under gentle shaking (100 rpm) at room temperature. Seeds immersed in sterile distilled water served as the untreated control. After treatment, six seeds from each condition were transferred into sterile containers containing solidified 0.7% (w/v) agarose gel as a gelling matrix. Containers were maintained in a controlled-growth incubator at 25 °C ± 1°C, 95%–100% relative humidity, and a 16-h light/8-h dark photoperiod for 14 days. Following incubation, seedlings were harvested for the evaluation of growth parameters and biochemical indices associated with plant vigor.

### 2.9 Pot experiment for growth promotion and disease suppression in *O. sativa* L.

A pot experiment was conducted to evaluate the plant growth-promoting and biocontrol potential of the most active bacterial isolate, P3, against *B. oryzae* in *O. sativa* L. (rice). Plastic pots were each filled with 500 g of sterilized potting substrate composed of peat, sand, and perlite (2:1:1, v/v/v). Rice seeds were divided into two main groups: the control, with seeds soaked in sterile distilled water, and the bacterial treatment group, with seeds soaked in a P3 suspension (1 × 10^7^ CFU·mL^−1^) for 48 h before sowing. Each main group was further subdivided into two subgroups: one left uninfected and the other challenged with the fungal pathogen.

Pathogen inoculation was performed at the 14-day seedling stage by spraying 10 mL of a *B. oryzae* spore suspension (1 × 10^7^ spores·mL^−1^) evenly over each pot. Immediately after inoculation, plants were maintained under high relative humidity (approximately 100%) for 48 h by covering the pots with transparent polyethylene bags to facilitate conidial germination and successful infection. This experimental design comprised four treatments: i) untreated and uninfected control seedlings, ii) seeds treated only with the P3 isolate, iii) uninoculated seeds infected with *B. oryzae* (INF), and iv) seeds treated with the P3 isolate followed by fungal infection (P3 + INF).

All treatments were arranged in a completely randomized design (CRD) with three replicates per treatment, and each replicate consisted of four seedlings per pot (n = 4). Plants were maintained under controlled growth conditions at 25 °C ± 2°C with a 16-h light/8-h dark photoperiod and a relative humidity of 95%–100%. Irrigation was carried out using sterile distilled water as needed. After 21 days of growth, seedlings were harvested for morphological, physiological, and biochemical analyses.

### 2.10 Physiological and biochemical analyses of rice seedlings

To evaluate the effects of P3 inoculation and *B. oryzae* infection on rice physiology, a series of morphological, photosynthetic, oxidative, and antioxidant parameters were assessed in 21-day-old seedlings. These analyses were conducted using standardized spectrophotometric and colorimetric assays to quantify changes in growth, pigment composition, oxidative biomarkers, and both enzymatic and non-enzymatic antioxidant defense systems. All measurements were performed in triplicate with independent biological replicates.

#### 2.10.1 Growth parameters and total soluble carbohydrates

Seedlings were gently washed with distilled water to remove adhering debris, and shoot and root lengths were measured using a digital caliper. Fresh weight (FW) was recorded immediately after blotting excess moisture, while dry weight (DW) was determined after oven-drying the seedlings at 50°C for 72 h to a constant weight. Total soluble carbohydrate content was determined using the phenol–sulfuric acid method described by Masuko et al. (2005), with minor modifications. Briefly, 0.1 mL of the aqueous extract obtained from finely ground dry leaf powder was mixed with 0.5 mL of 5% (w/v) phenol solution, followed by the rapid addition of 2.5 mL of concentrated H_2_SO_4_. The reaction mixture was incubated at 30°C for 30 min to develop a stable yellow–orange chromophore, and absorbance was measured at 490 nm using a UV–VIS spectrophotometer (Shimadzu UV-1800, Japan).

#### 2.10.2 Photosynthetic pigment analysis

Photosynthetic pigments, including chlorophyll *a* (Chl *a*), chlorophyll *b* (Chl *b*), and total carotenoids, were quantified spectrophotometrically using the methods described by Arnon (1949) and Lichtenthaler and Wellburn (1983), with slight modifications. Fresh leaf tissues (0.1 g) were homogenized in 10 mL of 80% (v/v) acetone using a pre-chilled mortar and pestle. After extraction in the dark at 4 °C for 10 hours, the extract was centrifuged at 10,000 *× g* for 10 min at 4°C to obtain a clear supernatant. The absorbance of the supernatant was measured at 663, 645, and 470 nm using a UV–VIS spectrophotometer (Shimadzu UV-1800, Japan). Pigment concentrations were calculated using the following equations and expressed as mg·g^−1^ FW:


$$\text{Chl a}=12.7(A_{663})-2.69(A_{645})$$

$$\text{Chl b}=22.9(A_{645})-4.68(A_{663})$$

$$\text{Carotenoids}=(1000A_{470}–3.27\text{Chl}\;a–104\text{Chl }b)/229$$

#### 2.10.3 Analysis of oxidative stress biomarkers

Hydrogen peroxide (H_2_O_2_) and malondialdehyde (MDA) contents were quantified as indicators of oxidative stress using the methods described by Velikova et al. (2000) and Heath and Packer (1968), respectively, with minor modifications to standardize the procedures. For H_2_O_2_ quantification, 0.1 g of fresh leaf tissue was homogenized in 5 mL of ice-cold 0.1% (w/v) trichloroacetic acid (TCA) and centrifuged at 12,000 *× g* for 15 min at 4°C. The reaction mixture contained 0.5 mL of the supernatant, 0.5 mL of 10 mM potassium phosphate buffer (pH 7.0), and 1 mL of 1 M potassium iodide (KI). Absorbance was measured at 390 nm using a UV–VIS spectrophotometer (Shimadzu UV-1800, Japan). For MDA estimation, 0.1 g of fresh leaf tissue was homogenized in 5 mL of 5% (w/v) TCA and centrifuged at 10,000 *× g* for 10 min. To 2 mL of the resulting supernatant, 2 mL of 0.67% (w/v) thiobarbituric acid (TBA) prepared in 20% (w/v) TCA was added. The mixture was heated at 95°C for 20 min in a water bath and then rapidly cooled on ice to terminate the reaction. The absorbance of the supernatant was measured at 532 and 600 nm.

#### 2.10.4 Determination of non-enzymatic antioxidants

Total phenolic content (TPC) was determined using the Folin–Ciocalteu method (Jindal and Singh, 1975). An aliquot (0.2 mL) of the ethanolic extract was mixed with 1 mL of 10% (v/v) Folin–Ciocalteu’s reagent and 0.8 mL of 7.5% (w/v) Na_2_CO_3_ solution. The mixture was incubated for 60 min at room temperature in the dark, and absorbance was measured at 650 nm. Gallic acid (0–1 mg·mL^−1^) was used to prepare the calibration curve (R^2^ = 0.9948). The limit of detection was 0.02 mg·mL^−1^. Results were expressed as mg gallic acid equivalents (GAE)·g^−1^ DW. The total flavonoid content (TFC) was measured according to the method described by Chang et al. (2002). The assay mixture consisted of 0.5 mL of ethanolic extract, 0.1 mL of 10% (w/v) AlCl_3_, 0.1 mL of 1 M potassium acetate, and 4.3 mL of 95% ethanol. After incubation for 30 min at room temperature, the absorbance was recorded at 417 nm. Quercetin (0–1 mg·mL^−1^) served as the standard for calibration (calibration curve R^2^ = 0.8677). The limit of detection for the TFC was 0.05 mg·mL^−1^, and the results were expressed as mg quercetin equivalents (QE)·g^−1^ DW. Ascorbic acid content was quantified according to Omaye et al. (1979). Fresh leaf tissue (0.1 g) was homogenized in 5% (w/v) sulfosalicylic acid and centrifuged at 10,000 *× g* for 10 min. The reaction mixture consisted of 1.5 mM Na_2_HPO_4_, 0.15 N H_2_SO_4_, 2% (w/v) ammonium molybdate, and 0.5 mL of supernatant. The mixture was incubated at 60°C for 40 min and then cooled to room temperature. Absorbance was then measured at 660 nm. A standard calibration curve of l-ascorbic acid (0–5 mg·mL^−1^, R^2^ = 0.9956) was used for quantification, and results were expressed as mg ascorbic acid equivalents·g^−1^ FW. Total antioxidant capacity (TAC) was assessed using the phosphomolybdenum method (Prieto et al., 1999). The reagent was prepared by mixing 0.6 M H_2_SO_4_, 28 mM Na_2_HPO_4_, and 4 mM ammonium molybdate in a 1:1:1 ratio. The reaction mixture consisted of 1 mL of TAC reagent and 100 µL of ethanolic extract, which was incubated at 95°C for 90 min and then cooled to room temperature. Absorbance was read at 765 nm. Ascorbic acid (0–4 mg·mL^−1^) was used as the reference standard (calibration curve R^2^ = 0.9912). The limit of detection for TAC was 0.03 mg·mL^−1^, and results were expressed as mg ascorbic acid equivalents·g^−1^ DW. All spectrophotometric measurements were performed in triplicate, and results were presented as mean ± standard error.

#### 2.10.5 Extraction and determination of antioxidant enzymatic activities

Fresh leaves of *O. sativa* L. were used to determine the activity of key antioxidant enzymes, namely, polyphenol oxidase (PPO), peroxidase (POD), ascorbate peroxidase (APX), and phenylalanine ammonia-lyase (PAL). Enzyme extraction was performed by homogenizing 0.5 g of fresh leaf tissue in 5 mL of ice-cold 50 mM potassium phosphate buffer (pH 7.0) containing 1% (w/v) polyvinylpyrrolidone (PVP) and 0.1 mM EDTA. The homogenate was centrifuged at 12,000 *× g* for 15 min at 4°C, and the resulting supernatant was used as the enzyme source. All enzyme assays were performed in triplicate, and enzymatic activities were expressed as units (U)·mg^−1^ protein, where one unit was defined as a 0.01 change in absorbance per minute under the assay conditions. Blank reactions without enzyme extract were used as controls for calibration. Enzymatic activities were standardized to total soluble protein content determined by the Bradford assay (Bradford, 1976).

PAL activity was determined following Lim et al. (1997). The reaction mixture [total volume 3 mL contained 0.5 mL enzyme extract, 100 mM Tris–HCl buffer (pH 8.8), and 40 mM l-phenylalanine]. The mixture was incubated at 37°C for 60 min, and the reaction was terminated by adding 0.2 mL of 4 M HCl. Formation of *trans*-cinnamic acid was quantified spectrophotometrically at 290 nm using a molar extinction coefficient of 9,630 M^−1^·cm^−1^. PPO activity was assayed according to the method of Kumar and Khan (1982). The reaction mixture contained 2 mL of 0.1 M phosphate buffer (pH 6.5), 0.5 mL of 2 mM pyrogallol, and 0.5 mL of enzyme extract. The reaction was incubated at 25°C for 10 min and stopped by adding 1 mL of 2.5 N H_2_SO_4_. The formation of purpurogallin was measured at 420 nm, and activity was expressed as the rate of change in absorbance per minute. POD activity was determined using the method of Kato and Shimizu (1987). The assay mixture contained 1.9 mL of 100 mM potassium phosphate buffer (pH 5.8), 1 mL of 7.2 mM guaiacol, 1 mL of 11.8 mM H_2_O_2_, and 0.1 mL of enzyme extract. The increase in absorbance due to tetraguaiacol formation was recorded at 470 nm for 3 min, using a molar extinction coefficient of 26.6 mM^−1^·cm^−1^. APX activity was measured following Nakano and Asada (1981). The 3-mL reaction mixture consisted of 50 mM sodium phosphate buffer (pH 7.0), 0.1 mM H_2_O_2_, 0.1 mM EDTA, 0.5 mM ascorbic acid, and 0.1 mL of enzyme extract. The decline in absorbance at 290 nm, corresponding to ascorbate oxidation, was monitored for 3 min using a molar extinction coefficient of 2.8 mM^−1^·cm^−1^.

### 2.11 Quantification of the relative expression of plant resilience-related genes

Total RNA was extracted from fresh leaves of *O. sativa* L. using the RNeasy Mini Kit (Qiagen, Germantown, MD, USA) following the manufacturer’s instructions after homogenizing the tissue under liquid nitrogen using a pre-chilled mortar and pestle. RNA purity and integrity were verified by measuring the A260/A280 ratio and by agarose gel electrophoresis. First-strand cDNA was synthesized from 1 µg of total RNA using the SuperScript™ III Reverse Transcriptase Kit (Invitrogen, Carlsbad, California, USA) according to the manufacturer’s protocol. Quantitative real-time PCR (qRT-PCR) was conducted on a Rotor-Gene 6000 system (Qiagen/ABI, Foster City, California, USA) using SYBR Green PCR Master Mix (Thermo Fisher Scientific, Waltham, Massachusetts, USA). Each 20-µL reaction contained 10 µL of SYBR Green Master Mix, 0.5 µL each of forward and reverse primers (10 µM), 2 µL of cDNA template, and nuclease-free water to a final volume of 20 µL. The amplification program consisted of an initial denaturation at 95°C for 10 min, followed by 40 cycles of denaturation (95°C, 15 s), annealing [58–60°C, 30 s; depending on the melting temperature (T_m_) of each primer pair], and extension (72°C, 30 s). A melt curve analysis was performed at the end of the run to confirm the specificity of amplification. Gene-specific primers for the selected stress-resilience genes are listed in **Supplementary Table S2**. The housekeeping gene β-actin was used as the internal reference to normalize expression data. All reactions were conducted in triplicate to ensure reproducibility. Relative expression levels of the target genes were calculated using the 2^−ΔΔCT^ method (Livak and Schmittgen, 2001).

### 2.12 Statistical analysis

All data were expressed as the mean ± standard error (SE) of three independent biological replicates. Statistical analyses were performed using one-way analysis of variance (ANOVA) to assess the significance of treatment effects. Mean separations were conducted using Duncan’s multiple-range test (DMRT) at a significance level of *p* < 0.05. All analyses were carried out using the XLSTAT software (version 2014.5.03; Addinsoft, Paris, France).

## 3 Results

### 3.1 Isolation, screening, and functional characterization of rhizospheric isolates

Four morphologically distinct bacterial isolates (P1–P4) were obtained from the rhizosphere of *P. australis* on KBA, NA, and TSA media. These isolates were screened for their key biocontrol and plant growth-promoting traits, including HCN production, IAA synthesis, phosphate solubilization, and biofilm formation (**Table 1**). The results revealed considerable variability among the isolates in all tested parameters. HCN production, a marker of antifungal potential, showed significant (*p* < 0.05) differences among isolates. Isolate P3 exhibited the highest HCN production (0.081 ± 0.0020), whereas P1 produced the lowest value of 0.023 ± 0.0017. These results suggest that isolates P3 and P4 possess stronger antagonistic potential than the other isolates.

**Table 1:** Biocontrol- and plant growth-promoting traits of bacterial isolates obtained from the rhizosphere of *Phragmites australis*.

Bacterial isolates	Phosphate solubilization zone (mm)	Hydrogen cyanide (HCN; Optical density (OD) value)	Indole-3-acetic acid (IAA; mg·L^−1^)	Biofilm formation
P1	10.66 ± 0.3^c^	0.023 ± 0.0017^c^	0.38 ± 0.007^d^	0.14 ± 0.0031^d^
P2	14.7 ± 0.2^b^	0.031 ± 0.0014^c^	0.42 ± 0.008^c^	0.57 ± 0.0030^b^
P3	19.5 ± 0.36^a^	0.081 ± 0.0020^a^	0.86 ± 0.011^a^	0.61 ± 0.0057^a^
P4	18.4 ± 0.33^a^	0.068 ± 0.0023^b^	0.80 ± 0.001^b^	0.38 ± 0.0032^c^

Values represent the mean ± standard error (n = 3). Distinct letters within each column indicate statistically significant differences among isolates (*p* < 0.05) according to one-way ANOVA followed by Duncan’s multiple-range test. Phosphate solubilization was assessed on Pikovskaya’s agar. HCN production was quantified as OD_625_ from the picrate–carbonate assay. IAA concentration was determined colorimetrically using Salkowski’s reagent. Biofilm formation was evaluated using the microtiter plate assay at OD_570_.

Similarly, the production of IAA—a key phytohormone that influences root elongation and nutrient uptake—was the highest in isolate P3 (0.86 ± 0.011 mg·L^−1^), whereas isolates P2 and P1 showed markedly lower levels (0.42 and 0.38 mg·L^−1^, respectively). The ability of these isolates to produce substantial amounts of IAA highlights their potential to enhance rice seedling growth and early root development. Phosphate-solubilization assays demonstrated a clear advantage for isolates P3 and P4, which exhibited the largest solubilization zones (19.5 ± 0.36 and 18.4 ± 0.33 mm, respectively), indicating superior efficiency in mobilizing insoluble phosphate forms. By contrast, isolate P1 formed the smallest halo (**Table 1**), suggesting comparatively limited phosphate-solubilizing activity.

Regarding biofilm formation, which enhances rhizosphere colonization and pathogen suppression, P3 again exhibited the highest optical density (OD_570_ = 0.61 ± 0.0057), whereas isolates P4 and P1 formed significantly weaker biofilms (**Table 1**). Collectively, these results clearly identify isolate P3 as the most potent strain, combining strong biocontrol-related traits with superior plant growth-promoting activities. Preliminary biosafety assays showed that P3 was non-hemolytic, coagulase-negative, and sensitive to all tested antibiotic classes (**Supplementary Figure S2**; **Supplementary Table 1**). Sensitivity to β-lactams, cephalosporins, aminoglycosides, quinolones, macrolides, and monobactams indicates the absence of broad-spectrum antibiotic resistance determinants. These traits, together with their environmental origin, suggest a low-risk, biosafety level 1 (BSL-1) profile suitable for controlled-environment experimentation. These findings justify its selection for subsequent greenhouse and molecular analyses aimed at elucidating its role in enhancing rice growth and resistance to *B. oryzae*.

### 3.2 Characterization of bacterial and fungal isolates

Among the four bacterial isolates obtained from the rhizosphere of *P. australis*, isolate P3 exhibited distinctive morphological and biochemical characteristics consistent with the *Stutzerimonas* group. Colonies were smooth, creamy to light-brown, and non-fluorescent on KBA plates. Cells were Gram-negative, rod-shaped, and motile, exhibiting catalase- and oxidase-positive reactions (**Supplementary Figure S1**). SEM confirmed the bacilliform morphology typical of *S. stutzeri*, and the isolate also produced a melanin-like, diffusible pigment on NA plates (**Supplementary Figure S1**). These phenotypic traits, combined with molecular identification, confirmed the isolate’s identity as *S. stutzeri* AUMC B-503. The fungal pathogen isolated from rice leaves exhibiting brown spot symptoms produced grayish-brown to dark-brown colonies with a velvety texture on PDA (**Supplementary Figure S3**). Microscopic examination revealed dark, geniculate conidiophores bearing curved, multicellular conidia with three to seven septa (**Supplementary Figure S3**). These conidial features are diagnostic of *B. oryzae*, and the morphological identification was further confirmed by ITS-based molecular characterization.

**Figure 1:**
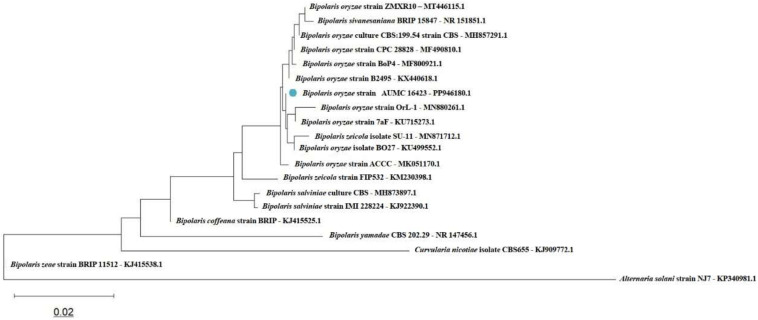
Phylogenetic tree illustrating the placement of *Bipolaris oryzae* strain AUMC 16423 among closely related taxa, based on internal transcribed spacer (ITS) sequence alignment. The tree was constructed using the neighbor-joining method with 1,000 bootstrap replicates. Branch lengths are proportional to evolutionary distances calculated using the Maximum Composite Likelihood model, with the scale bar indicating 0.02 substitutions per nucleotide site.

**Figure 2:**
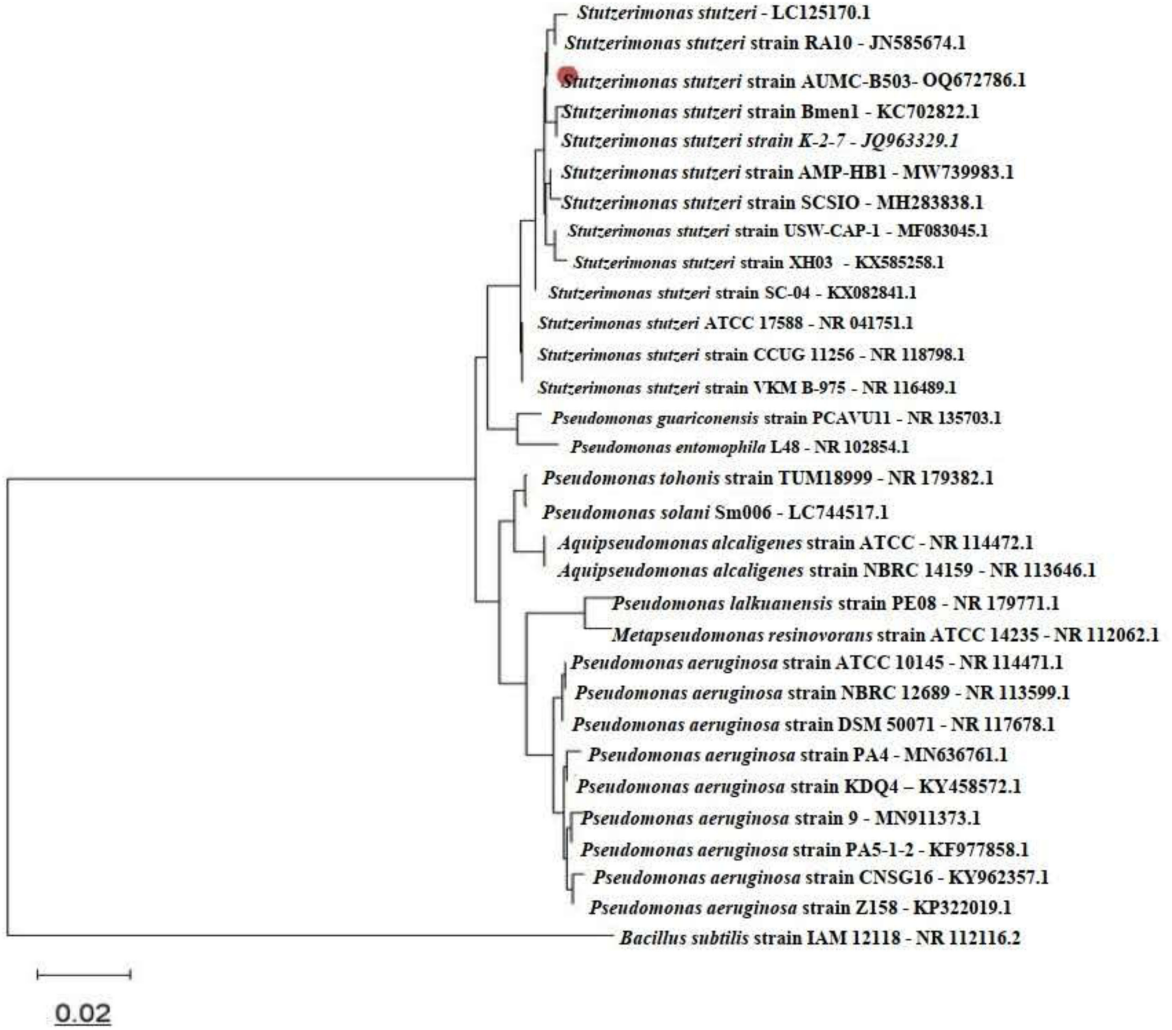
Phylogenetic tree depicting the position of *Stutzerimonas stutzeri* strain AUMC B-503 among closely related taxa based on 16S rRNA gene sequences. The evolutionary history was inferred using the neighbor-joining method with 1,000 bootstrap replicates. The tree is drawn to scale, with branch lengths representing evolutionary distances calculated using the Maximum Composite Likelihood method. Evolutionary distances are expressed as the number of base substitutions per site (scale bar = 0.02).

The ITS region was successfully amplified and sequenced, yielding a dataset comprising 20 fungal taxa and 793 aligned nucleotide positions. In the resulting NJ phylogenetic tree (**Figure 1**), *B. oryzae* strain AUMC 16423 (GenBank accession no. PP946180.1) clustered within a well-supported monophyletic group representing the *B. oryzae* complex. Closely related taxa included *Bipolaris zeicola*, whereas *Bipolaris salviniae* and *Bipolaris coffeana* formed adjacent sister clades. More distantly related species, such as *Bipolaris yamadae* and *Curvularia nicotiae*, appeared at the basal position of the Pleosporales lineage, with *Alternaria solani* (family Pleosporaceae) serving as the designated outgroup. The tree topology and high bootstrap support values (≥70%) corroborated the current taxonomic placement within the genus *Bipolaris* and confirmed the species-level identification of the pathogen. Similarly, phylogenetic analysis based on 16S rRNA gene sequencing verified the bacterial isolate as *S. stutzeri* (formerly *P. stutzeri*). The bacterial dataset comprised 31 taxa and 1,556 aligned nucleotide sites (**Figure 2**). The strain *S. stutzeri* AUMC B-503 (GenBank accession no. OQ672786.1) formed a distinct, well-supported cluster within the *S. stutzeri*/*P. stutzeri* complex, clearly separated from the *Pseudomonas aeruginosa* group and other representative members of the family Pseudomonadaceae, including *Pseudomonas guariconensis*, *Pseudomonas tohonis*, *Aquipseudomonas alcaligenes*, and *Metapseudomonas resinovorans*. *Bacillus subtilis* strain IAM 12118 was used as the outgroup. The greater total branch length of the bacterial tree compared with that of the bacterial dataset indicated higher genetic diversity within the family Pseudomonadaceae. Overall, both phylogenies exhibited clear taxonomic resolution, providing strong molecular evidence for the accurate identification of the fungal pathogen *B. oryzae* and the bacterial antagonist AUMC B-503, and the corresponding sequences have been deposited in the GenBank database under accession numbers PP946180 and OQ672786, respectively.

### 3.3 Antifungal activity of bacterial isolates against *B. oryzae*

**Figure 3:**
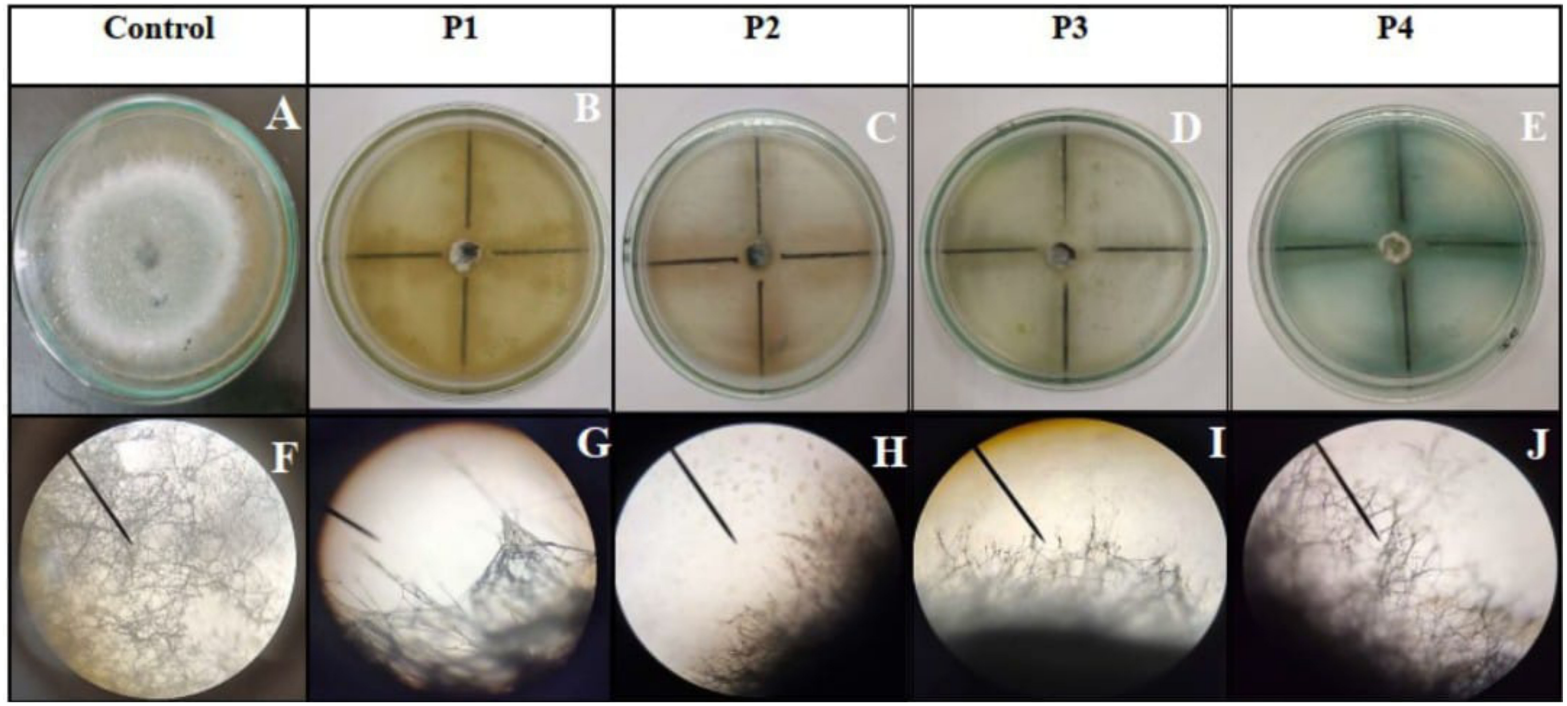
Antifungal activity of bacterial isolates (P1–P4) against *Bipolaris oryzae* AUMC 16423. **(A)** Control plate showing unrestricted fungal growth. **(B–E)** Dual culture assays with bacterial isolates P1–P4, respectively, displaying distinct inhibition zones. **(F)** Control fungal hyphae. **(G–J)** Hyphal morphology following co-culture with P1–P4 under light microscopy (×40 magnification) showing hyphal distortion, fragmentation, and cytoplasmic lysis induced by bacterial antagonism.

All four isolates (P1–P4) exhibited strong antifungal activity against *B. oryzae* AUMC 16423 in the dual-culture assays, as shown in **Figures 3A–E**. The degree of mycelial growth inhibition differed significantly among isolates (*p* < 0.05), as determined by one-way ANOVA followed by DMRT. The inhibition percentages were 83.4% ± 0.2%, 86.0% ± 0.1%, 87.9% ± 0.3%, and 85.5% ± 0.2% for isolates P1, P2, P3, and P4, respectively. Among these, isolate P3 (AUMC B-503) exhibited the highest antagonistic potential, producing a distinct inhibition zone around the fungal colony and restricting radial growth by nearly 88%. Microscopic observations provided further insight into the underlying antifungal mechanism (**Figures 3F–J**). The untreated control showed healthy, elongated, and intact fungal hyphae (**Figure 3F**), whereas *B. oryzae* hyphae co-cultured with the bacterial isolates exhibited severe morphological deformities. These included hyphal shrinkage, fragmentation, and cytoplasmic disintegration, indicating the potent activity of antifungal metabolites. Such structural damage suggests that extracellular metabolites produced by the bacterial isolates—such as HCN and diffusible antibiotics—interfered with fungal cell wall integrity and hyphal elongation. Collectively, these findings confirm that isolate P3, identified as *S. stutzeri* AUMC B-503, exhibits the strongest antifungal activity among the tested isolates, supporting its selection for subsequent greenhouse trials as a promising biocontrol agent against *B. oryzae*.

### 3.4 Effects of bacterial isolates on the growth and biochemical composition of rice seedlings in the agarose assay

**Figure 4:**
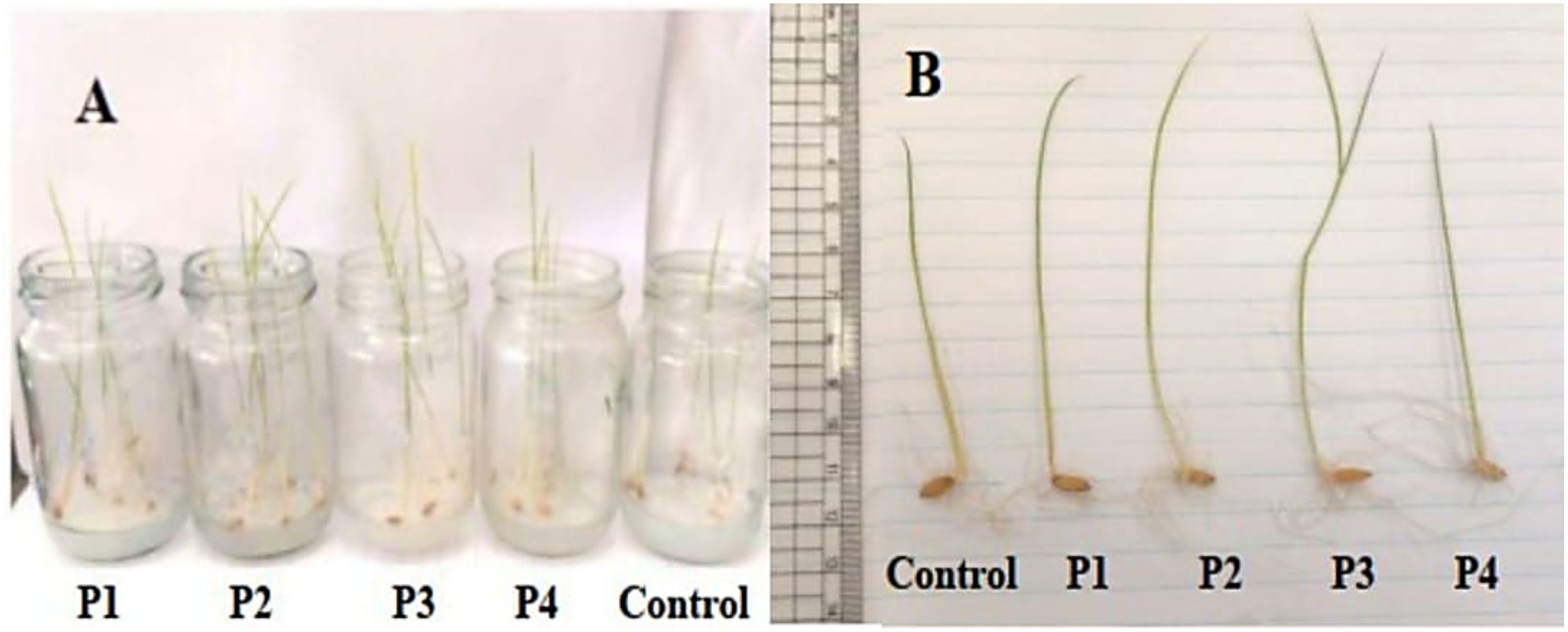
Growth promotion of rice seedlings by bacterial isolates in agarose medium. **(A)** Rice seedlings grown in glass jars containing 0.7% agarose, inoculated with P1–P4, compared with the uninoculated control. **(B)** Comparative seedling morphology showing enhanced shoot and root elongation in bacteria-treated plants (P1–P4) relative to the control.

All four isolates (P1–P4) significantly enhanced the early growth performance of rice seedlings compared with the uninoculated control (**Table 2**, **Figure 4**). Inoculated seedlings exhibited marked increases in shoot and root elongation, as well as in FW and DW accumulation. The degree of growth stimulation varied significantly among isolates (*p* ≤ 0.05), with P3 producing the most pronounced effects. Seedlings treated with P3 achieved shoot and root lengths of 11.13 ± 0.27 cm and 10.20 ± 0.60 cm, respectively, representing approximately 120% and 96% increases relative to the control. FW and DW also increased by 47% and 43%, respectively, indicating a substantial enhancement of overall seedling vigor.

**Table 2:** Growth parameters and total soluble carbohydrates of rice seedlings treated with bacterial isolates (P1–P4).

Treatment	Shoot length (cm)	Root length (cm)	Fresh weight (g/plant)	Dry weight (g/plant)	Total soluble carbohydrates (mg·g^−1^ DW)	Amino acid (mg·g^−1^ DW)
Control	5.0 ± 0.57^c^	5.2 ± 0.98^c^	0. 59 ± 0. 01^c^	0.14 ± 0.00^c^	675.2 ± 10.4^b^	1.89 ± 0.37^b^
P1	8.23 ± 0.31^b^	7.5 ± 0.58^b^	0.70 ± 0. 01^b^	0. 16 ± 0.001^b^	704.6 ± 3.4^ab^	2.49 ± 0.37^b^
P2	9.4 ± 0.36^ab^	8.3 ± 0.52^ab^	0. 81 ± 0. 01^ab^	0.19 ± 0.002^ab^	709.8 ± 9.7^ab^	3.50 ± 0.24^ab^
P3	11.13 ± 0.27^a^	10.2 ± 0.60^a^	0. 87 ± 0. 03^a^	0.20 ± 0.007^a^	719.1 ± 8.0^a^	4.85 ± 0.67^a^
P4	9.6 ± 0.50^ab^	7.6 ± 0.84^b^	0. 74 ± 0.014^b^	0.17 ± 0.009^b^	700.7 ± 7.2^ab^	2.32 ± 0.29^b^

Data are means ± SE (n = 5). Different letters indicate significant differences (*p* ≤ 0.05) according to Duncan’s multiple-range test.

DW, dry weight.

Biochemical analyses further revealed that bacterial inoculation significantly increased total soluble carbohydrate and free amino acid contents compared with the control. Total soluble carbohydrate levels ranged from 704.6 ± 3.4 (P1) to 719.1 ± 8.0 mg·g^−1^ DW (P3), whereas total free amino acids increased to 4.85 ± 0.67 mg·g^−1^ DW in P3-treated plants—more than double the control value. Statistical analysis confirmed that P3 differed significantly from all other treatments (*p* ≤ 0.05), whereas P2 and P4 exhibited intermediate effects (**Table 2**). These results demonstrate that rhizosphere-derived bacterial isolates possess plant growth-promoting potential under sterile *in vitro* conditions, with AUMC B-503 exhibiting the strongest capacity to stimulate rice seedling development and metabolic activity. The enhanced accumulation of carbohydrates and amino acids suggests improved photosynthetic efficiency and nitrogen assimilation in response to bacterial inoculation.

### 3.5 Modulation of oxidative stress biomarkers and non-enzymatic antioxidant defenses in rice seedlings

**Figure 5:**
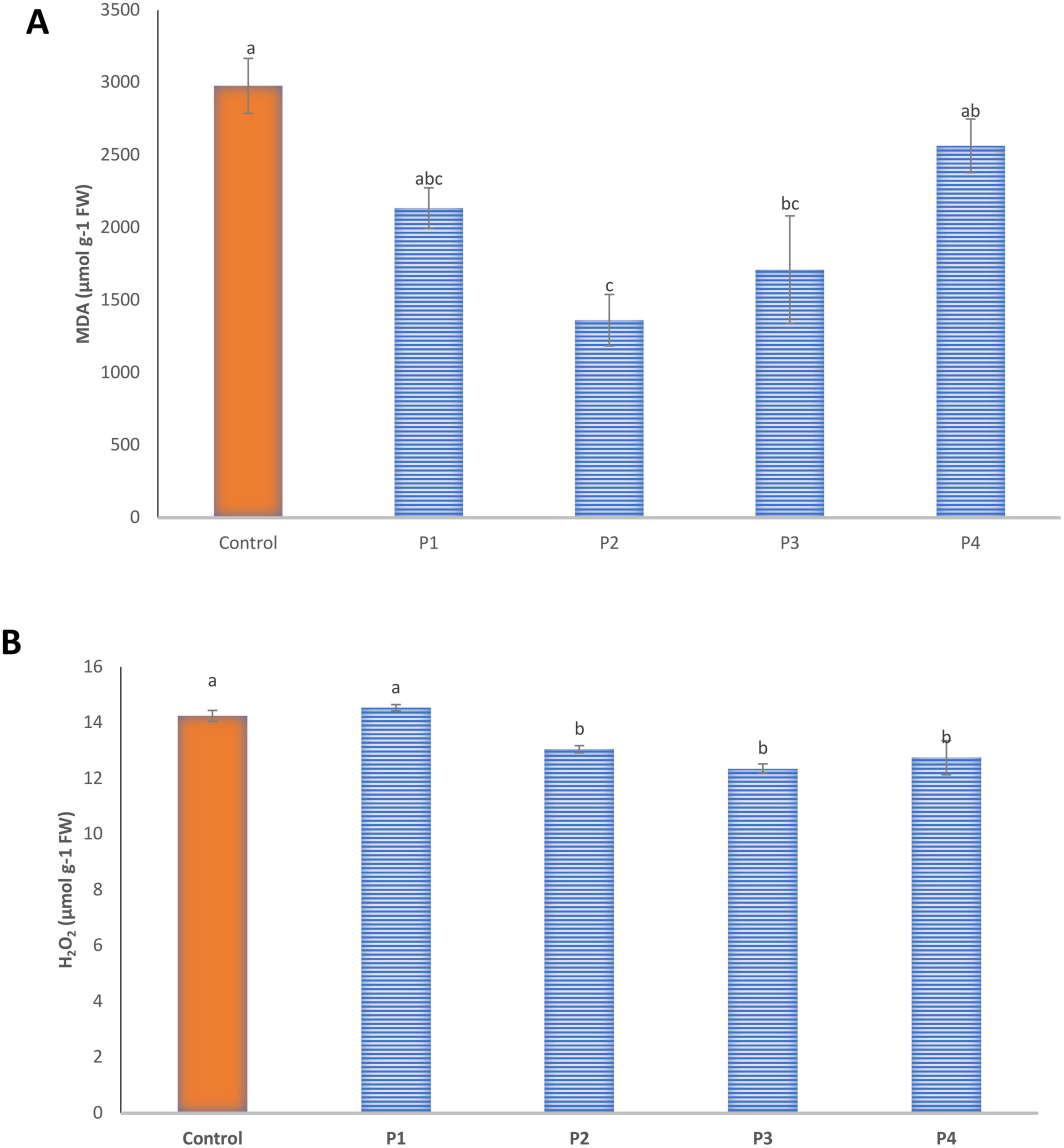
Effect of bacterial inoculation on oxidative stress biomarkers in rice seedlings. Bars represent **(A)** malondialdehyde (MDA) and **(B)** hydrogen peroxide (H_2_O_2_) contents in leaves of seedlings treated with isolates P1–P4 compared with the uninoculated control. Values are means ± SE (n = 3). Columns marked with different letters differ significantly at *p* ≤ 0.05.

The inoculation of rice seedlings with the four bacterial isolates (P1–P4) resulted in a marked reduction of oxidative stress compared with the uninoculated control (**Figure 5**). The levels of MDA and H_2_O_2_—key indicators of lipid peroxidation and oxidative damage—were significantly reduced in all inoculated treatments (*p* ≤ 0.05). Among the isolates, P3 (AUMC B-503) produced the most pronounced decrease, with MDA and H_2_O_2_ concentrations of 1,362.45 and 12.35 µmol·g^−1^ FW, respectively, representing reductions of approximately 54% and 13% relative to the control. These findings indicate that bacterial inoculation effectively alleviated oxidative damage, likely through enhanced antioxidant activity. Consistent with this observation, the levels of non-enzymatic antioxidants, including total phenolics, flavonoids, l-ascorbic acid, and TAC, were significantly elevated in bacterially treated seedlings, particularly in those inoculated with P3 (**Table 3**). P3 treatment increased total phenolics to 221.4 ± 5.5 mg·g^−1^ DW, flavonoids to 74.7 ± 0.5 mg·g^−1^ DW, l-ascorbic acid to 384.1 ± 9.6 mg·g^−1^ FW, and TAC to 11.6 ± 1.1 mg ascorbic acid equivalents·g^−1^ DW, representing 1.5- to 1.6-fold increases over control values. These increases suggest that *Stutzerimonas*-mediated stimulation of antioxidant metabolism reinforces cellular redox balance, limiting membrane lipid peroxidation and hydrogen peroxide accumulation. Together, these results confirm that AUMC B-503 is the most effective isolate for improving rice seedling tolerance to oxidative stress by simultaneously reducing reactive oxygen species (ROS)-related damage and activating non-enzymatic antioxidant defenses.

**Table 3:** Non-enzymatic antioxidant contents in rice seedlings inoculated with bacterial isolates (P1–P4).

Treatment	Total phenolics (mg·g^−1^ DW)	Total flavonoids (mg·g^−1^ DW)	l-Ascorbic acid (mg·g^−1^ FW)	Total antioxidant capacity (TAC; mg ASA·g^−1^ DW)
P1	149.7 ± 1.1^bc^	58.5 ± 0.7^bc^	322.4 ± 10.2^b^	9.0 ± 0.08^ab^
P2	169.9 ± 3.2^b^	63.5 ± 1.7^b^	335.1 ± 8.9^ab^	9.8 ± 0.13^ab^
P3	221.4 ± 5.5^a^	74.7 ± 0.5^a^	384.1 ± 9.6^a^	11.6 ± 1.1^a^
P4	162.6 ± 1.6^bc^	55.3 ± 1.4^c^	311.6 ± 16.1^b^	9.3 ± 0.25^b^
Control	142.4 ± 8.2^c^	43.4 ± 0.4^d^	316.5 ± 8.5^b^	7.3 ± 0.23^b^

Values are means ± SE (n = 3). Different letters indicate significant differences (*p* ≤ 0.05) according to Duncan’s multiple-range test.

DW, dry weight; FW, fresh weight.

### 3.6 Physiological and biochemical responses of rice plants in the pot experiment

To evaluate the overall plant growth-promoting and stress-alleviating effects of AUMC B-503 under pathogen challenge, a range of physiological and biochemical parameters were analyzed in rice seedlings subjected to the different treatments. The assessed parameters included growth and biomass traits, photosynthetic pigment contents, oxidative stress biomarkers, and both enzymatic and non-enzymatic antioxidant components.

#### 3.6.1 Growth parameters and total soluble carbohydrate contents

**Figure 6:**
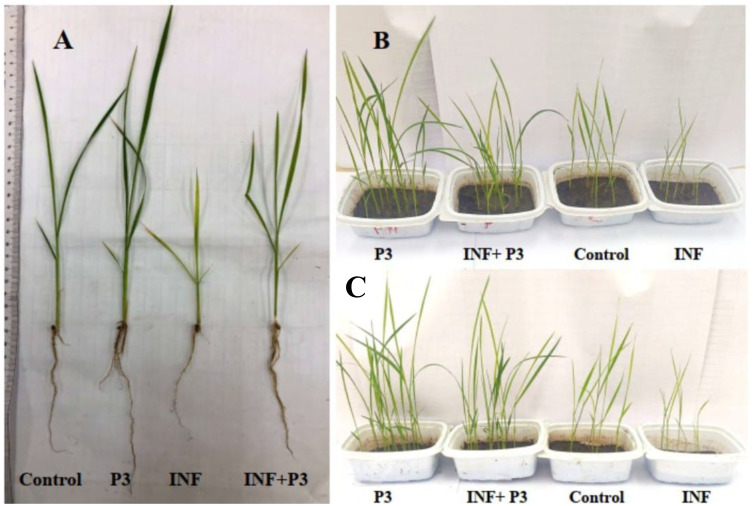
Effects of *Stutzerimonas stutzeri* AUMC B-503 (P3) on rice seedling growth under normal and *Bipolaris oryzae* infection conditions after 21 days in the pot experiment. **(A)** Representative seedlings from each treatment. **(B, C)** Overall appearance of rice plants showing improved shoot and root development in bacterized plants compared with uninoculated and infected seedlings. Control, untreated and uninfected control seedlings; INF, uninoculated seeds infected with *Bipolaris oryzae*; P3, seeds treated with AUMC B-503 only; P3 + INF, seeds treated with AUMC B-503 followed by fungal infection.

In the pot experiment, the inoculation of rice seedlings with P3 (AUMC B-503) significantly enhanced overall growth and biomass accumulation compared with both the uninoculated control and the pathogen-infected group. As shown in **Figure 6A**, seedlings treated with AUMC B-503 exhibited visibly longer shoots and more developed root systems than either control or infected plants. Similarly, the side-by-side comparison in **Figures 6B, C** illustrates that AUMC B-503-treated plants displayed more vigorous and uniform growth. In contrast, infection by *B. oryzae* (INF) markedly suppressed plant height and root elongation. Quantitative measurements confirmed these visual observations (**Table 4**). Pathogen infection alone caused a pronounced reduction in shoot and root growth—by approximately 21.5% and 30.3%, respectively—along with significant declines in fresh and dry biomass as well as total soluble carbohydrate content. Bacterization with AUMC B-503 resulted in significant (*p* ≤ 0.05) increases in shoot length, root length, FW, DW, and total soluble carbohydrate content—by 33.3%, 30.3%, 148.2%, 61.0%, and 19.7%, respectively—compared with the untreated control (**Table 4**). Moreover, in the combined treatment (P3 + INF), AUMC B-503 markedly mitigated the inhibitory effects of the fungal pathogen. Bacterized–infected plants exhibited 54.5%, 54.3%, 123.9%, 104.0%, and 57.5% higher shoot length, root length, FW, DW, and carbohydrate content, respectively, than infected plants without bacterial inoculation. These results demonstrate that AUMC B-503 not only promotes rice growth under normal conditions but also mitigates the adverse effects of *B. oryzae* infection, likely by enhancing nutrient uptake efficiency and stimulating carbohydrate metabolism to sustain energy supply under pathogen stress.

**Table 4:** Growth parameters and total soluble carbohydrate contents of rice seedlings in the pot experiment.

Treatment	Shoot length (cm)	Root length (cm)	Fresh weight (g/plant)	Dry weight (g/plant)	Carbohydrates (mg·g^−1^ DW)
Control	19.50 ± 1.04^b^	11.00 ± 1.0^ab^	0.145 ± 0.01^bc^	0.039 ± 0.002^b^	322.6 ± 28.4^a^
INF	15.30 ± 1.42^c^	7.66 ± 1.3^b^	0.096 ± 0.01^c^	0.022 ± 0.001^c^	192.2 ± 11.9^b^
P3	27.16 ± 0.44^a^	14.33 ± 0.92^a^	0.360 ± 0.02^a^	0.064 ± 0.008^a^	329.05 ± 18.8^a^
P3 + INF	23.70 ± 0.72^ab^	11.80 ± 1.5^ab^	0.215 ± 0.01^ab^	0.045 ± 0.004^b^	306.0 ± 34.4^ab^

Values represent the mean ± SE (n = 3). Different letters within a column indicate significant differences (*p* ≤ 0.05) according to Duncan’s multiple-range test.

Control, untreated and uninfected control seedlings; INF, uninoculated seeds infected with *Bipolaris oryzae*; P3, seeds treated with AUMC B-503 only; P3 + INF, seeds treated with AUMC B-503 followed by fungal infection; DW, dry weight.

#### 3.6.2 Photosynthetic pigment composition

The photosynthetic pigment profile of rice plants showed marked enhancement following inoculation with P3 (AUMC B-503) under both normal and infection-stress conditions (**Table 5**). Bacterized plants exhibited significant increases in Chl *a*, Chl *b*, and carotenoid contents compared with untreated controls, with improvements of 22.5%, 23.5%, and 54.5%, respectively. These increases indicate that bacterial inoculation enhanced pigment biosynthesis and stability, thereby supporting greater photosynthetic efficiency under optimal growth conditions. Infection with *B. oryzae* alone resulted in a pronounced decline in pigment content, with sharp reductions in both chlorophylls and carotenoids, reflecting pathogen-induced chloroplast disruption and oxidative stress. However, plants treated with AUMC B-503 before infection (P3 + INF) displayed substantially higher pigment levels than infected non-bacterized plants. The combined treatment increased Chl *a*, Chl *b*, and total carotenoids by 82.6%, 196%, and 47%, respectively, relative to infected seedlings. This significant recovery suggests that bacterial inoculation mitigated the adverse effects of fungal infection on chloroplast integrity and photosynthetic capacity, likely through the activation of antioxidant defense mechanisms and improved nutrient assimilation.

**Table 5:** Contents of photosynthetic pigments in leaves of rice seedlings treated with *Stutzerimonas stutzeri* AUMC B-503 under different treatments.

Treatment	Chlorophyll *a* (mg·g^−1^ FW)	Chlorophyll *b* (mg·g^−1^ FW)	Carotenoid (mg·g^−1^ FW)
Control	1.447 ± 0.118^a^	0.844 ± 0.037^ab^	0.421 ± 0.002^b^
INF	0.806 ± 0.116^b^	0.423 ± 0.063^b^	0.320 ± 0.036^b^
P3	1.773 ± 0.100^a^	1.043 ± 0.06^ab^	0.650 ± 0.098^a^
P3 + INF	1.473 ± 0.17^a^	1.252 ± 0.363^a^	0.470 ± 0.054^ab^

Values represent the mean ± SE (n = 3). Different letters within a column indicate significant differences (*p* ≤ 0.05) according to Duncan’s multiple-range test.

Control, untreated and uninfected control seedlings; INF, uninoculated seeds infected with *Bipolaris oryzae*; P3, seeds treated with AUMC B-503 only; P3 + INF, seeds treated with AUMC B-503 followed by fungal infection; FW, fresh weight.

#### 3.6.3 Oxidative stress biomarkers in the pot experiment

**Figure 7:**
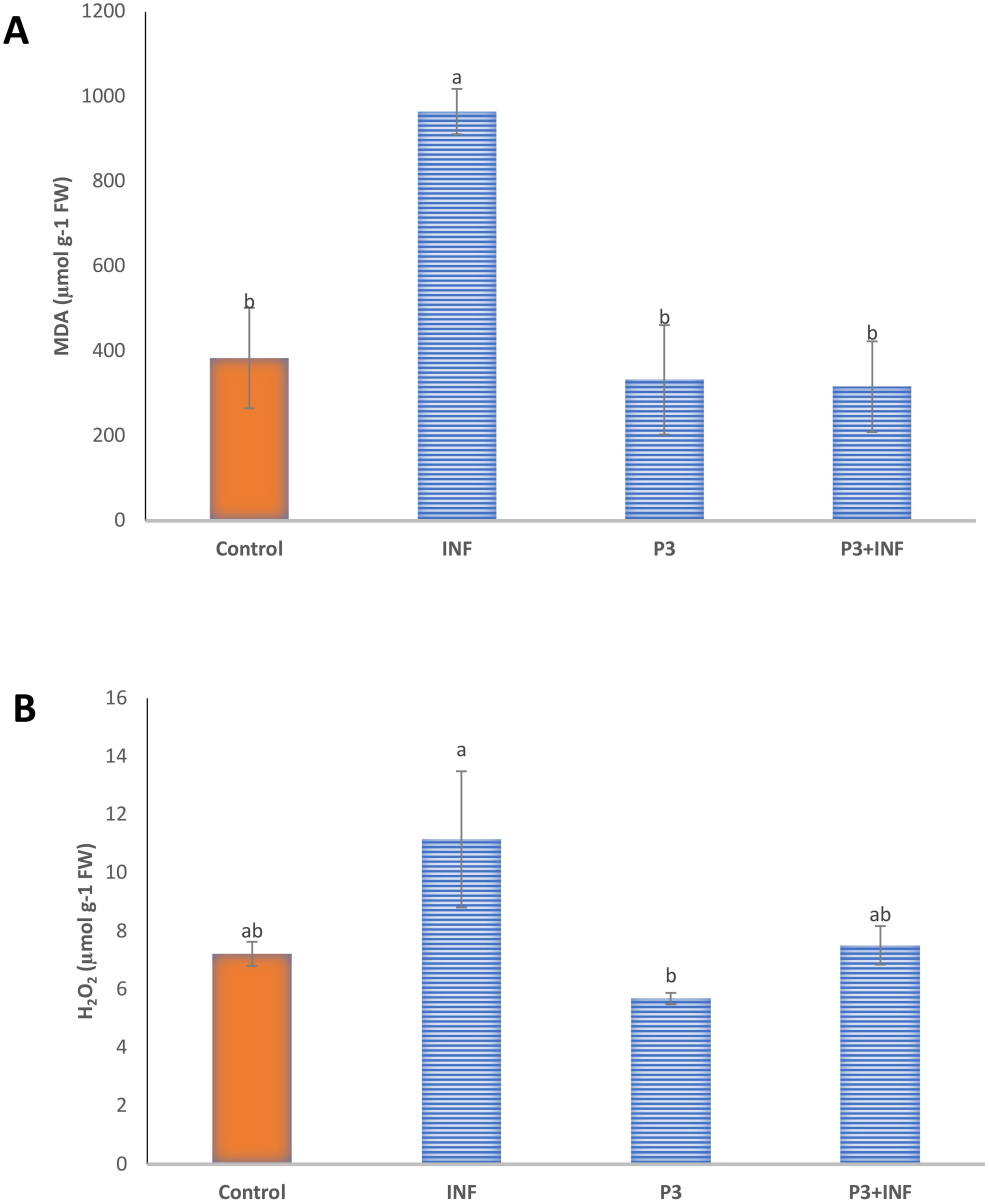
Oxidative stress biomarkers of rice seedlings in the pot experiment. **(A)** Malondialdehyde (MDA) and **(B)** hydrogen peroxide (H_2_O_2_) contents in rice leaves. Control, untreated and uninfected control seedlings; INF, uninoculated seeds infected with *Bipolaris oryzae*; P3, seeds treated with AUMC B-503 only; P3 + INF, seeds treated with AUMC B-503 followed by fungal infection. Data represent means ± SE (n = 3). Different letters above bars indicate significant differences (*p* ≤ 0.05) according to Duncan’s multiple-range test.

Oxidative stress levels in rice plants were assessed by quantifying MDA and H_2_O_2_ contents, which serve as indicators of lipid peroxidation and ROS accumulation, respectively (**Figure 7**). Fungal infection caused a marked rise in oxidative stress, with infected plants exhibiting 151.5% and 54.4% higher MDA and H_2_O_2_ contents, respectively, compared with the healthy control. This substantial increase reflects severe membrane lipid damage and excessive ROS generation induced by *B. oryzae* infection (**Figure 7**, INF panels). In contrast, rice seedlings inoculated with AUMC B-503 (P3 treatment) exhibited significantly lower (*p* ≤ 0.05) MDA and H_2_O_2_ levels than both the control and infected seedlings, indicating reduced oxidative stress and improved cellular redox balance. Notably, co-inoculation with AUMC B-503 and *B. oryzae* (P3 + INF) resulted in a pronounced decline in both biomarkers relative to infected plants, restoring MDA and H_2_O_2_ concentrations close to those of the uninfected control. These results demonstrate that AUMC B-503 effectively mitigates pathogen-induced oxidative damage, likely through the activation of antioxidant defense mechanisms and stabilization of cellular membrane integrity.

#### 3.6.4 Non-enzymatic antioxidant content in the pot experiment

**Figure 8:**
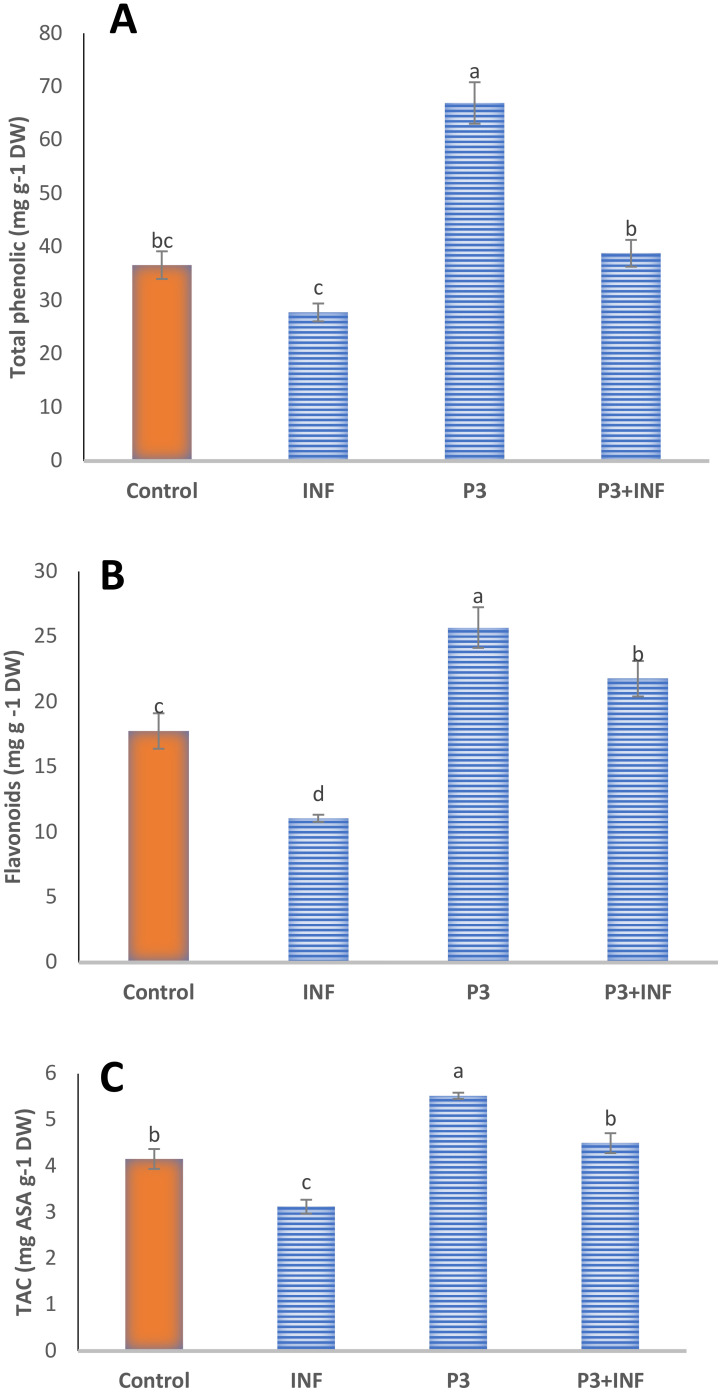
Levels of non-enzymatic antioxidants in rice seedlings during the pot experiment. **(A)** Total phenolic content (mg·g^−1^ DW), **(B)** total flavonoids (mg·g^−1^ DW), and **(C)** total antioxidant capacity (TAC; mg Ascorbic Acid (ASA)·g^−1^ DW). Control, untreated and uninfected control seedlings; INF, uninoculated seeds infected with *Bipolaris oryzae*; P3, seeds treated with AUMC B-503 only; P3 + INF, seeds treated with AUMC B-503 followed by fungal infection. Values are means ± SE (n = 3). Different letters indicate significant differences (*p* ≤ 0.05) according to Duncan’s multiple-range test. DW, dry weight.

The non-enzymatic antioxidant system of rice seedlings showed marked enhancement in response to AUMC B-503 inoculation under both normal and pathogen-stress conditions (**Figure 8**). Relative to the untreated control, plants treated with AUMC B-503 exhibited significant (*p* ≤ 0.05) increases in total phenolic compounds, total flavonoids, and TAC by 82%, 44.6%, and 33%, respectively. These findings indicate that bacterial inoculation stimulates the biosynthesis of phenolic and flavonoid compounds—major contributors to the non-enzymatic antioxidant defense system that protects cellular structures from oxidative damage. Infection by *B. oryzae* (INF) substantially reduced the levels of these metabolites compared with controls, reflecting oxidative depletion and suppression of secondary metabolism under pathogen stress (**Figure 8**). However, plants subjected to the combined treatment (P3 + INF) exhibited a pronounced recovery of antioxidant capacity. Compared with infected seedlings, AUMC B-503 treatment increased total phenolics, flavonoids, and TAC by 39.1%, 96.8%, and 44%, respectively. This pronounced recovery under infection stress suggests that AUMC B-503 induces the accumulation of protective metabolites, thereby strengthening the redox buffering system and enhancing plant stress tolerance.

#### 3.6.5 Antioxidant enzymatic activities in the pot experiment

The activities of key antioxidant enzymes, including PPO, POD, PAL, and APX, were significantly enhanced in rice plants treated with AUMC B-503 compared to the control (**Table 6**). Specifically, enzymatic activities in the bacterized seedlings increased by 25.8% (PPO), 22.6% (POD), 43.3% (PAL), and 51.8% (APX), demonstrating that bacterial inoculation stimulates the plant’s enzymatic antioxidant machinery. Fungal infection with *B. oryzae* markedly suppressed all four enzymatic activities, reflecting oxidative damage and a compromised defense system in the infected plants. However, the co-inoculation of rice plants with AUMC B-503 and the pathogen (P3 + INF) substantially restored enzymatic activities. In comparison to infected seedlings, the combined treatment resulted in significant increases of 40.5%, 53.8%, 60.6%, and 56.9% in PPO, POD, PAL, and APX activities, respectively. These enhancements indicate that AUMC B-503 effectively reactivates ROS-scavenging enzymes and phenolic biosynthetic pathways under biotic stress. Collectively, the elevated levels of both enzymatic and non-enzymatic antioxidants confirm that AUMC B-503 elicits a broad-spectrum defense response in rice, enhancing its resilience against *B. oryzae* infection.

**Table 6:** Activities of antioxidant enzymes in rice seedlings under different treatments.

Treatment	Polyphenol oxidase (PPO; µM·g^−1^ FW·min^−1^)	Peroxidase (POD; µM·g^−1^ FW·min^−1^)	Phenylalanine ammonia-lyase (PAL; µM·g^−1^ FW·min^−1^)	Ascorbate peroxidase (APX; µM·g^−1^ FW·min^−1^)
Control	0.0969 ± 0.004^b^	1.192 ± 0.062^ab^	0.754 ± 0.009^bc^	0.0145 ± 0.007^ab^
INF	0.0755 ± 0.001^c^	0.771 ± 0.080^b^	0.578 ± 0.006^b^	0.0068 ± 0.0006^b^
P3	0.122 ± 0.011^a^	1.461 ± 0.226^a^	1.08 ± 0.096^a^	0.0220 ± 0.002^ab^
P3 + INF	0.106 ± 0.004^ab^	1.187 ± 0.039^ab^	0.928 ± 0.072^ab^	0.0457 ± 0.0095^a^

Values are means ± SE (n = 3). Different letters within a column indicate significant differences (*p* ≤ 0.05) according to Duncan’s multiple-range test.

Control, untreated and uninfected control seedlings; INF, uninoculated seeds infected with *Bipolaris oryzae*; P3, seeds treated with AUMC B-503 only; P3 + INF, seeds treated with AUMC B-503 followed by fungal infection; FW, fresh weight.

### 3.7 *S. stutzeri* AUMC B-503 promotes the expression of plant resilience genes

**Figure 9:**
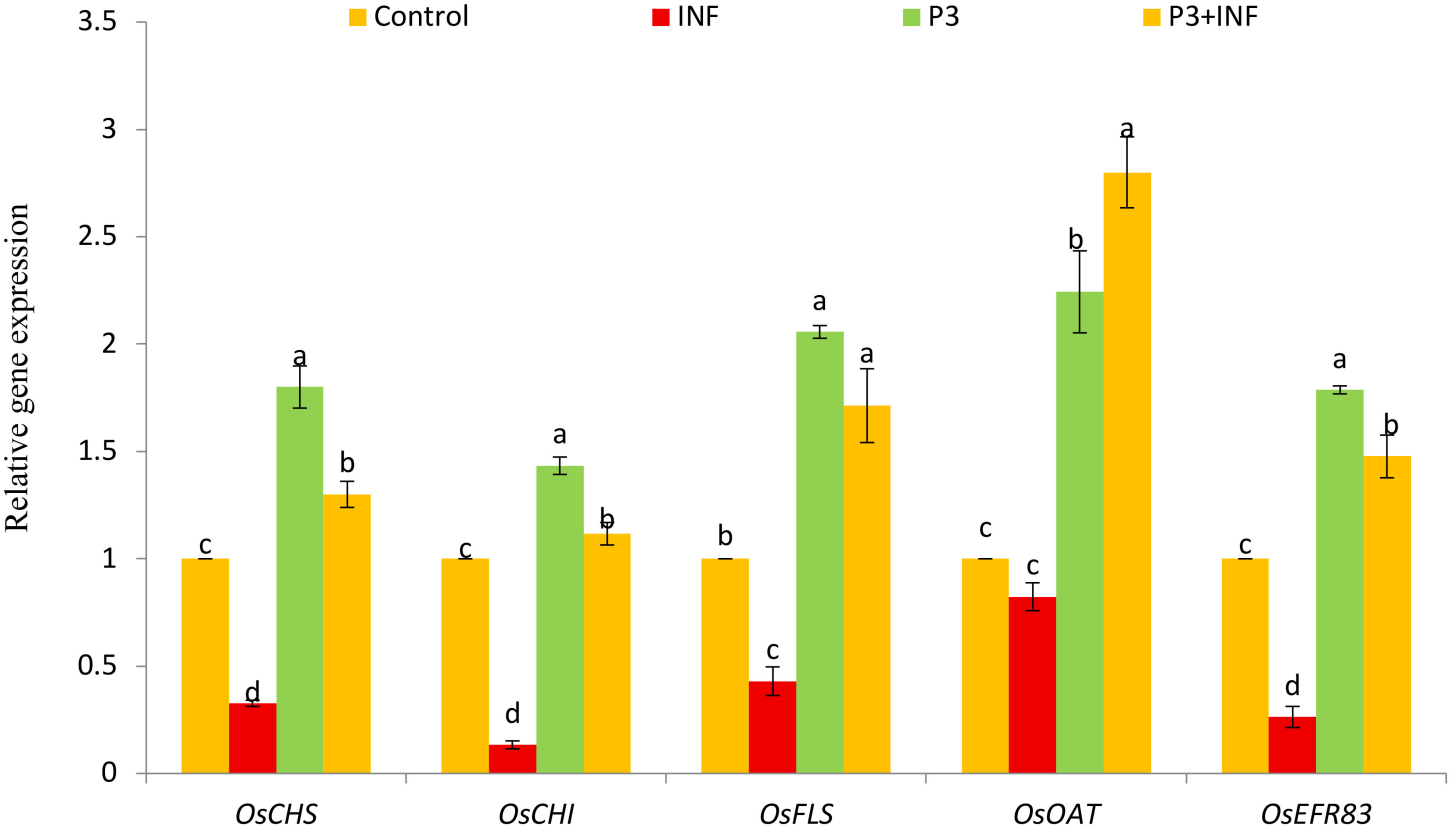
Relative expression of stress-responsive and developmental genes (*OsCHS*, *OsCHI*, *OsFLS*, *OsOAT*, and *OsERF83*) in rice seedlings. Control, untreated and uninfected control seedlings; INF, uninoculated seeds infected with *Bipolaris oryzae*; P3, seeds treated with AUMC B-503 only; P3 + INF, seeds treated with AUMC B-503 followed by fungal infection. Data represent means ± SE (n = 3). Different letters indicate significant differences (*p* ≤ 0.05) according to Duncan’s multiple-range test.

To elucidate the molecular basis of the enhanced stress tolerance observed in treated rice plants, the expression profiles of five stress- and development-related genes—*OsCHS*, *OsCHI*, *OsFLS*, *OsOAT*, and *OsERF83*—were examined (**Figure 9**). Compared with the control, *B. oryzae*-infected seedlings exhibited a marked downregulation of *OsCHS*, *OsCHI*, *OsFLS*, and *OsERF83*, reflecting the pathogen-induced suppression of defense and secondary-metabolism pathways. Conversely, inoculation with AUMC B-503 significantly upregulated all five genes relative to the control, indicating the activation of both phenylpropanoid and stress response pathways. The most pronounced induction occurred in *Stutzerimonas*-treated and infected seedlings (P3 + INF), where expression levels of *OsCHS*, *OsCHI*, *OsFLS*, *OsOAT*, and *OsERF83* increased by approximately 1.3-, 1.11-, 1.7-, 2.8-, and 1.47-fold, respectively, compared with the control (**Figure 9**). This coordinated upregulation suggests that AUMC B-503 enhances the transcriptional activation of key genes involved in flavonoid biosynthesis (*OsCHS*, *OsCHI*, and *OsFLS*), osmoprotectant metabolism (*OsOAT*), and ethylene-responsive signaling (*OsERF83*), thereby strengthening the plant’s defense network and overall resilience to fungal stress.

## 4 Discussion

Brown spot disease, caused by *B. oryzae*, remains one of the most destructive fungal diseases affecting rice, resulting in substantial yield losses and reductions in grain quality worldwide (Kaboré et al., 2025). Conventional chemical fungicides are often effective but pose environmental and health risks, underscoring the need for sustainable biological alternatives (Gikas et al., 2022). PGPR represent promising tools for eco-friendly crop protection and yield enhancement, owing to their ability to promote plant growth, suppress pathogens, and trigger systemic resistance (Hasan et al., 2024). In this study, rhizobacterial isolate P3, obtained from the rhizosphere of *P. australis*, was evaluated for its biocontrol potential and plant growth-promoting effects in rice. The combined morphological, biochemical, microscopic, and molecular characteristics provide strong evidence supporting the accurate identification of both isolates. The bacterial isolate exhibited typical *S. stutzeri* features (Lalucat et al., 2006; Özen and Ussery, 2012). Similarly, the fungal isolate displayed grayish to dark colonies with curved, multicellular, septate conidia, which are diagnostic features of *B. oryzae* (Manamgoda et al., 2012). These morphological and microscopic observations were entirely consistent with the phylogenetic placement based on ITS and 16S rRNA analyses, confirming the species-level identity of *B. oryzae* AUMC 16423 and *S. stutzeri* AUMC B-503. Together, these integrated datasets reinforce the robustness of the taxonomic conclusions and address potential concerns regarding reliance solely on sequence-based identification.

Multilocus Sequence Analysis (MLSA) and genome-based taxonomy provide superior resolution for distinguishing closely related bacterial and fungal species; however, 16S rRNA and ITS sequencing remain internationally accepted and reliable molecular markers for initial species-level identification in plant-associated microbial studies where genomic data are not available (Beattie, 2006; Manamgoda et al., 2012). In the present analysis, both markers produced high bootstrap support and consistent phylogenetic clustering with verified reference strains, confirming their suitability for accurate taxonomic placement. The phylogenetic trees generated in this study incorporated multiple reference and type strains to ensure reliable species-level comparison. The bacterial dataset comprised 31 taxa, while the fungal dataset contained 20 taxa. The clustering patterns observed in the phylogenetic trees demonstrate meaningful evolutionary and ecological relationships. The bacterial phylogenetic analysis revealed well-defined clustering patterns consistent with current taxonomic classifications. The *S. stutzeri* complex forms a distinct, strongly supported monophyletic clade positioned near the top of the tree, comprising multiple closely related strains (LC125170.1, RA10, AUMC OQ672786.1, Bmen1, and K-2-7) together with representative *S. stutzeri* isolates. This clade structure supports the recent taxonomic reclassification of several *P. stutzeri* lineages into the genus *Stutzerimonas* (Özen and Ussery, 2012). A second major clade encompassed *P. aeruginosa* strains, forming a robust monophyletic group that included isolates from diverse environmental and clinical origins (ATCC 10145, NBRC 12689, DSM 50071, PA4, KD04, strain 9, PAS-1-2, CNSG16, and Z158). The genetic cohesion of this clade, despite variation in sampling time and location, reflects the well-documented genomic stability of *P. aeruginosa* as a clinically significant pathogen (Stover et al., 2000). Several taxa, *P. guariconensis*, *Pseudomonas entomophila*, *P. tohonis*, *Pseudomonas solani*, *A. alcaligenes*, *Pseudomonas lalkuanensis*, and *M. resinovorans*, occupied intermediate phylogenetic positions, illustrating the evolutionary complexity within the Pseudomonadaceae family. *B. subtilis* IAM 12118 served as an appropriate outgroup, confirming the monophyly of Pseudomonadaceae. The grouping of *S. stutzeri* strains reflects genetic diversification within rhizosphere-adapted lineages, suggesting potential ecological specialization in plant-associated niches (Lalucat et al., 2006; Özen and Ussery, 2012). Similarly, the monophyletic clustering of *B. oryzae* isolates from various geographic origins supports the view that this pathogen forms a well-defined species complex exhibiting host adaptation and limited genetic divergence across rice-growing regions (Manamgoda et al., 2012). Also, the phylogenetic reconstruction of the *Bipolaris* genus similarly revealed intricate species-level relationships. A predominant cluster comprising *B. oryzae* strains formed a strongly supported monophyletic clade, with strain AUMC 16423 (GenBank PP946180.1) positioned within this group. This placement confirms its species identity and demonstrates a close genetic affinity with other *B. oryzae* isolates from diverse geographic origins (ZMXR10, BRIP 15847, CBS:198.54, CPC 28828, BoP4, and B2495). Fine-scale phylogenetic structuring was evident, with some strains (OrL-1 and 7aF) forming a subclade alongside *B. zeicola* SU-11 and *B. oryzae* BO27, suggesting potential cryptic diversity or recent speciation within the *Bipolaris* complex. The sister relationship between *B. zeicola* (FIP532) and the main *B. oryzae* cluster indicates a close evolutionary link, consistent with their overlapping host preferences and morphological similarities (Manamgoda et al., 2012). Distinct clades composed of *B. salviniae* and *B. coffeana* were recovered as sister groups to the *B. oryzae* complex; at the same time, more distantly related taxa such as *B. yamadae* and *C. nicotiae* occupied basal positions within the Pleosporales. *A. solani* served as the outgroup, validating the tree topology. In both phylogenetic trees, the branch-length scale of 0.02 substitutions per site indicates low intraspecific divergence within species complexes and substantially higher interspecific and intergeneric distances, providing strong molecular support for accurate taxonomic assignment of the studied isolates. Such relationships reinforce the accuracy of the molecular identification and provide insight into the evolutionary stability of both microbial taxa.

While several *Pseudomonas* spp. have been described as PGPR, studies specifically demonstrating the antifungal and defense-inducing capacities of *P. stutzeri* in rice remain scarce (Sandhya et al., 2010; Shen et al., 2013; Dorjey et al., 2017). The present work provides new evidence, supported by morphological, biochemical, and molecular analyses, demonstrating that AUMC B-503 acts as an effective biocontrol and growth-promoting strain. This contribution expands the current understanding of *P. stutzeri* diversity and activity, as previous studies have reported similar effects in maize (Gong et al., 2022) and wheat (Pandey et al., 2013). However, its defense-related and gene-regulatory roles in rice under *B. oryzae* stress remain largely unexplored.

The antagonistic activity exhibited by AUMC B-503 against *B. oryzae* is consistent with earlier reports describing the bacterial capacity to produce siderophores, HCN, IAA, and various lytic enzymes involved in pathogen suppression (Pandey et al., 2013; Saravanan et al., 2025). However, no biochemical or molecular assays such as GC–MS, LC–MS, or targeted gene expression analyses were performed in the present study to identify or quantify these metabolites. The results, therefore, indicate potential rather than experimentally confirmed antifungal mechanisms. Future work involving metabolomic profiling and transcriptomic or qPCR-based detection of antifungal biosynthetic genes will be essential to validate the compounds and pathways underlying the observed inhibition. This clarification aligns the current findings with recognized standards for mechanism validation in microbial biocontrol research. The growth-promoting and biocontrol potential of AUMC B-503 derives from a combination of plant growth-promoting traits, including IAA production, phosphate solubilization, HCN production, and biofilm formation. These attributes are characteristic of efficient PGPR that enhance nutrient uptake, stimulate root elongation, and improve overall plant vigor, while simultaneously suppressing phytopathogens (Pandey and Gupta, 2020; Ghadamgahi et al., 2022). The HCN produced by AUMC B-503 likely contributes to its antifungal effect against *B. oryzae* by interfering with the pathogen’s respiratory metabolism, consistent with previous findings that HCN-producing *Pseudomonas* spp. inhibit fungal pathogens by the disruption of the respiratory chain and the inhibition of key metalloenzymes, such as cytochrome oxidase (Fischer et al., 2013; Mehmood et al., 2023). Additionally, phosphate solubilization enhances phosphorus bioavailability, while IAA secretion promotes cell elongation and root development, thereby supporting robust plant growth (Selvakumar et al., 2015). Biofilm formation further enhances biocontrol efficacy by facilitating rhizosphere colonization, competitive exclusion of pathogens, and stimulation of plant systemic resistance (Ajijah et al., 2023). These observations align with earlier studies, which have shown that *Pseudomonas chlororaphis* from the rhizosphere of *Anemarrhena asphodeloides* and *Pseudomonas* strain YM6 from peanut and maize both exhibit strong antifungal activity against several phytopathogens (Liu et al., 2022; Gong et al., 2022). Collectively, AUMC B-503 demonstrates a dual functional capacity as a nutrient mobilizer and biocontrol agent, exerting antagonistic effects through multiple, complementary biochemical pathways.

*S. stutzeri* AUMC B-503 was selected for greenhouse pot experiments based on its lack of hemolytic activity and the absence of coagulase enzyme production. Coagulase is a polypeptide that binds and activates prothrombin, converting fibrinogen to fibrin and promoting plasma clotting, thereby enhancing bacterial persistence and pathogenicity in host tissues (McAdow et al., 2012). Many pathogenic bacteria also secrete soluble hemolysins capable of lysing red blood cells (Herlax and Bakas, 2002). Evaluation of hemolytic activity provides an indicator of potential virulence and pathogenic risk (Suleiman, 2024). Hemolytic factors are key virulence determinants that facilitate bacterial survival within host tissues, enable access to nutrients from erythrocytes, and help evade host immune defenses (Wong et al., 2016). The AUMC B-503 strain was γ-hemolytic (non-hemolytic), coagulase-negative, and sensitive to multiple clinical antibiotics, indicating low pathogenic potential and suggesting that it poses minimal risk to human health.

The growth-promoting effect of AUMC B-503 was evident in both the agarose and pot experiments, where treated seedlings exhibited significantly greater shoot and root lengths, biomass, and soluble carbohydrate contents compared with untreated or infected controls. These enhancements align with the responses reported for other *Pseudomonas* spp. in cereals such as corn and tomato (Qessaoui et al., 2019; Georgieva et al., 2023). The increase in Chl *a*, Chl *b*, and carotenoid contents observed in treated plants further indicates an improvement in photosynthetic efficiency and pigment stability, reflecting enhanced nitrogen assimilation and chloroplast functionality. Similar increases in chlorophyll content have been attributed to the auxin-mediated activation of photosynthetic genes and improved micronutrient uptake (Robas Mora et al., 2022; Ajijah et al., 2023). These combined physiological effects suggest that AUMC B-503 enhances primary metabolism and energy capture, thereby improving plant growth and stress resilience collectively.

Infection by *B. oryzae* typically induces oxidative stress in rice, characterized by the excessive accumulation of ROS, membrane lipid peroxidation, and elevated levels of MDA and H_2_O_2_ (Qessaoui et al., 2019; Morales and Munné-Bosch, 2024). The present findings demonstrate that AUMC B-503 mitigates this oxidative damage by reducing MDA and H_2_O_2_ accumulation, indicating effective ROS scavenging and improved redox balance. This stress attenuation can be attributed to both direct bacterial antioxidative activity and the stimulation of the plant’s own defense machinery. Similar findings have been reported in wheat and rice inoculated with PGPR, where enhanced enzymatic and non-enzymatic antioxidant systems suppressed oxidative stress markers (Pandey et al., 2013; Das and Roychoudhury, 2014). The significant increase in total phenolics, flavonoids, ascorbic acid, and total antioxidant capacity observed in bacterized seedlings further supports the activation of secondary metabolic pathways that protect plants from oxidative injury and pathogen invasion (Rudenko et al., 2023).

Moreover, AUMC B-503 enhanced the activities of PPO, POD, PAL, and APX—key enzymes involved in detoxifying ROS and reinforcing the plant cell wall. Enhanced PPO activity contributes to the oxidation of phenolic substrates into quinones, which possess antimicrobial properties (Queiroz et al., 2008). In contrast, PAL catalyzes the first step in the phenylpropanoid pathway, leading to lignin and flavonoid biosynthesis—critical components of induced systemic resistance (ISR) (Bi and Felton, 1995; Prasannath and De Costa, 2015). Elevated POD and APX activities indicate efficient H_2_O_2_ detoxification and redox regulation, consistent with the ISR responses observed in rice and wheat following PGPR treatment (Ashfaq et al., 2021; Rajendran et al., 2015). Together, these biochemical responses confirm that AUMC B-503 not only improves plant growth but also primes antioxidant defense systems against pathogen-induced oxidative stress.

At the molecular level, AUMC B-503 significantly upregulated the expression of *OsCHS*, *OsCHI*, *OsFLS*, *OsOAT*, and *OsERF83*, demonstrating its ability to modulate gene networks associated with flavonoid biosynthesis, osmoprotection, and stress signaling. The induction of *OsCHS*, *OsCHI*, and *OsFLS* was directly correlated with the observed accumulation of total flavonoids, suggesting that bacterial treatment enhances the transcriptional activation of the phenylpropanoid pathway, thereby strengthening cellular antioxidant capacity (Piasecka et al., 2015; Jatan et al., 2023). The significant upregulation of *OsOAT* indicates stimulation of the ornithine aminotransferase pathway, which contributes to proline biosynthesis—a well-known osmolyte and ROS scavenger (Gallie, 2013; Adamipour et al., 2020). Although proline content was not measured in this study, the observed *OsOAT* induction strongly suggests a role for proline metabolism in the stress tolerance elicited by AUMC B-503. Furthermore, the enhanced expression of *OsERF83*, a transcription factor in the ethylene-responsive factor family, suggests activation of ethylene and jasmonate cross-talk pathways that regulate pathogenesis-related genes and enhance disease resistance (Tezuka et al., 2019; Ma et al., 2024). Collectively, the combined biochemical and molecular evidence demonstrates that AUMC B-503 induces both enzymatic and transcriptional defense responses, integrating physiological and molecular mechanisms to promote plant resilience.

Overall, the findings of this study demonstrate that AUMC B-503 exerts a multifactorial mechanism combining antibiosis through HCN and biofilm formation, nutrient mobilization through phosphate solubilization and IAA production, and induced systemic resistance mediated by enhanced antioxidant enzymes and defense gene expression. This integrative mechanism highlights the strain’s potential as a dual-function PGPR that simultaneously enhances growth and confers disease resistance. While *S. stutzeri* has previously been reported for its biocontrol properties in maize, wheat, and peanut systems (Pandey et al., 2013; Gong et al., 2022), the present study provides new experimental evidence indicating that an AUMC B-503 strain from the *P. australis* rhizosphere can activate flavonoid- and ethylene-responsive defense pathways in rice. The integration of physiological, biochemical, and molecular analyses highlights the potential of AUMC B-503 as a sustainable biocontrol and biofertilization candidate for managing brown spot disease and enhancing rice performance under stress conditions.

Although AUMC B-503 exhibited strong antagonism against *B. oryzae*, several *S. stutzeri* lineages have been reported as opportunistic pathogens in humans (Lalucat et al., 2006). Therefore, preliminary biosafety screening was conducted. The isolate was non-hemolytic, coagulase-negative, and sensitive to all tested antibiotics, supporting its non-pathogenic character. Comparable features have been reported in biosafety level 1 *Pseudomonas* strains used in agriculture (Timmis et al., 2019). These characteristics meet the minimal biosafety requirements outlined by Sachana et al. (2025) and Mudgal et al. (2013) for low-risk microbial biocontrol agents. Nevertheless, comprehensive genome-based analyses targeting virulence and antimicrobial resistance genes, along with assessments of environmental persistence and potential non-target effects (on beneficial microbes, insects, and aquatic species), remain necessary before any field release (Sundh et al., 2011; O’Callaghan et al., 2022). Accordingly, AUMC B-503 is described here as a promising, low-risk biocontrol candidate under controlled conditions, pending further validation through genomic and ecological safety evaluations.

## 5 Conclusions

The present study demonstrated that *S. stutzeri* AUMC B-503, isolated from the rhizosphere of *P. australis*, possesses multiple plant growth-promoting and biocontrol traits that collectively enhance rice growth and resistance to *B. oryzae*. This bacterium exhibited strong phosphate solubilization, IAA and HCN production, and biofilm formation, which collectively contributed to improved nutrient uptake and pathogen suppression. Under both controlled and pathogen-stress conditions, AUMC B-503 significantly increased shoot and root growth, biomass, and photosynthetic pigment contents while reducing oxidative damage, as indicated by lower MDA and H_2_O_2_ levels. These physiological improvements were supported by enhanced activities of antioxidant enzymes (PPO, POD, PAL, and APX) and elevated levels of phenolics, flavonoids, and total antioxidant capacity, confirming the induction of a robust antioxidative defense system. At the molecular level, AUMC B-503 upregulated the expression of *OsCHS*, *OsCHI*, and *OsFLS*, correlating with increased flavonoid accumulation, and concurrently activated *OsOAT* and *OsERF83*, which are associated with proline metabolism and ethylene/jasmonate-mediated stress signaling, respectively. These transcriptional changes indicate that the bacterium modulates plant primary and secondary metabolism and primes systemic resistance through coordinated hormonal and antioxidant regulation. Overall, the findings suggest that *S. stutzeri* AUMC B-503 represents a promising, low-risk biocontrol and biofertilization candidate, characterized by the integration of physiological, biochemical, and transcriptional defense responses in rice against *B. oryzae*. Future work should focus on genome-based biosafety evaluation, non-target impact assessment, and regulatory validation to support its advancement toward field-scale application.

## Data Availability

The datasets presented in this study can be found in online repositories. The names of the repository/repositories and accession number(s) can be found in the article/**Supplementary Material**.

## Supplementary material

Supplementary Figure 1 Morphological and biochemical characterization of *Stutzerimonas stutzeri* AUMC B-503. **(A)** Gram-stained cells showing Gram-negative rod-shaped morphology. **(B)** Scanning electron micrograph (SEM) illustrating the bacillus-shaped cells at 3,500× magnification (scale bar = 5 µm). **(C)** Production of a melanin-like brown pigment on King’s B agar. **(D)** Positive catalase reaction evidenced by bubble formation upon exposure to hydrogen peroxide.

Supplementary Figure 2 Biosafety assays of *Stutzerimonas stutzeri* AUMC B-503. **(A)** Blood agar plate showing γ-hemolysis (non-hemolytic activity). **(B)** Negative coagulase reaction with absence of the clot formation. **(C)** Antibiotic susceptibility test showing clear inhibition zones around all tested antibiotic discs, indicating sensitivity to all tested antibiotics.

Supplementary Figure 3 Morphological characteristics of *Bipolaris oryzae* isolated from rice plants showing brown spot symptoms. **(A)** Colony morphology on potato dextrose agar (PDA) showing a grayish-olive, velvety surface with concentric ring pattern. **(B)** Microscopic view of dark, septate conidiophores producing brown, multiseptate, elliptical conidia (×1000 magnification).

Supplementary Table 1 Antibiotic classes, antimicrobial agents, and disc potencies used.

Supplementary Table 2 Primers used in this study.
