# Correction: Rhizosphere-derived *Stutzerimonas stutzeri* AUMC B-503: a promising biocontrol and plant growth-promoting strain for managing brown spot disease in rice (*Oryza sativa*)

**DOI:** 10.3389/fpls.2025.1765939

**Published:** 2026-01-02

**Authors:** Frontiers Production Office

**Affiliations:** Frontiers Media SA, Lausanne, Switzerland

**Keywords:** plant growth-promoting bacteria (PGPB), *Stutzerimonas stutzeri*, *Oryza sativa*, *Bipolaris oryzae*, *Phragmites australis*, biocontrol, sustainable agriculture

The affiliation for Reviewer Harekrushna Swain was erroneously given as “Eastern Regional Center, India”. The correction affiliation is “Botanical Survey of India, India”.

The original version of this article has been updated.

