# Supplementary material for: Rhizosphere-derived Stutzerimonas stutzeri AUMC B-503: a promising biocontrol and plant growth-promoting strain for managing brown spot disease in rice (Oryza sativa)
Source: Front Plant Sci. 2025 Dec 11;16:1700440. doi: 10.3389/fpls.2025.1700440 (PMC12738882; doi:10.3389/fpls.2025.1700440)
Supplement: Supplementary Figure 1 — Morphological and biochemical characterization of Stutzerimonas stutzeri AUMC B-503. (A) Gram-stained cells showing Gram-negative rod-shaped morphology. (B) Scanning electron micrograph (SEM) illustrating the bacillus-shaped cells at 3,500× magnification (scale bar = 5 µm). (C) Production of a melanin-like brown pigment on King’s B agar. (D) Positive catalase reaction evidenced by bubble formation upon exposure to hydrogen peroxide. [file DataSheet1.docx]

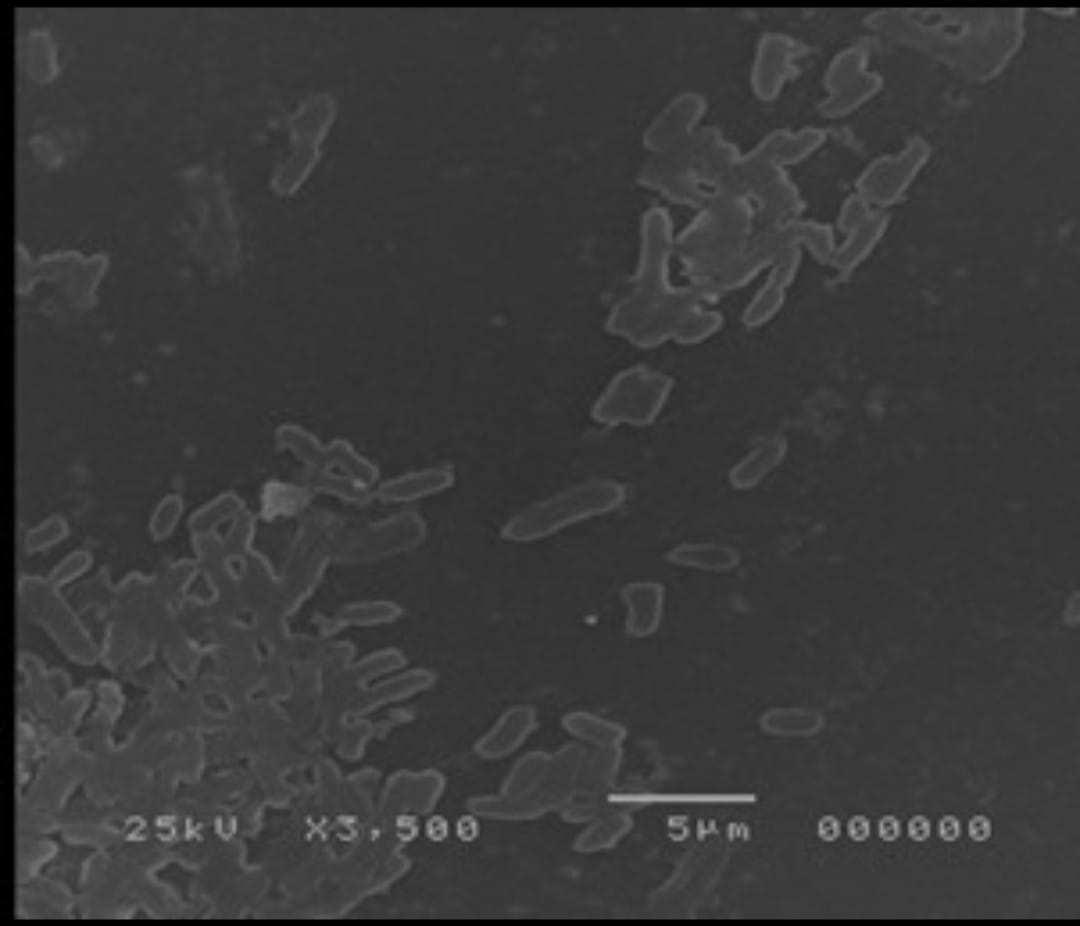

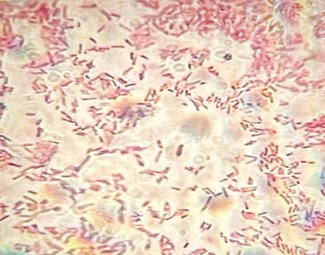

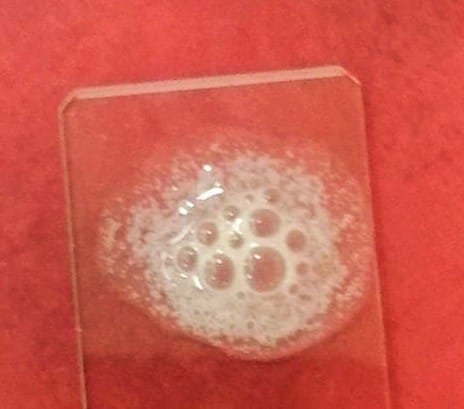

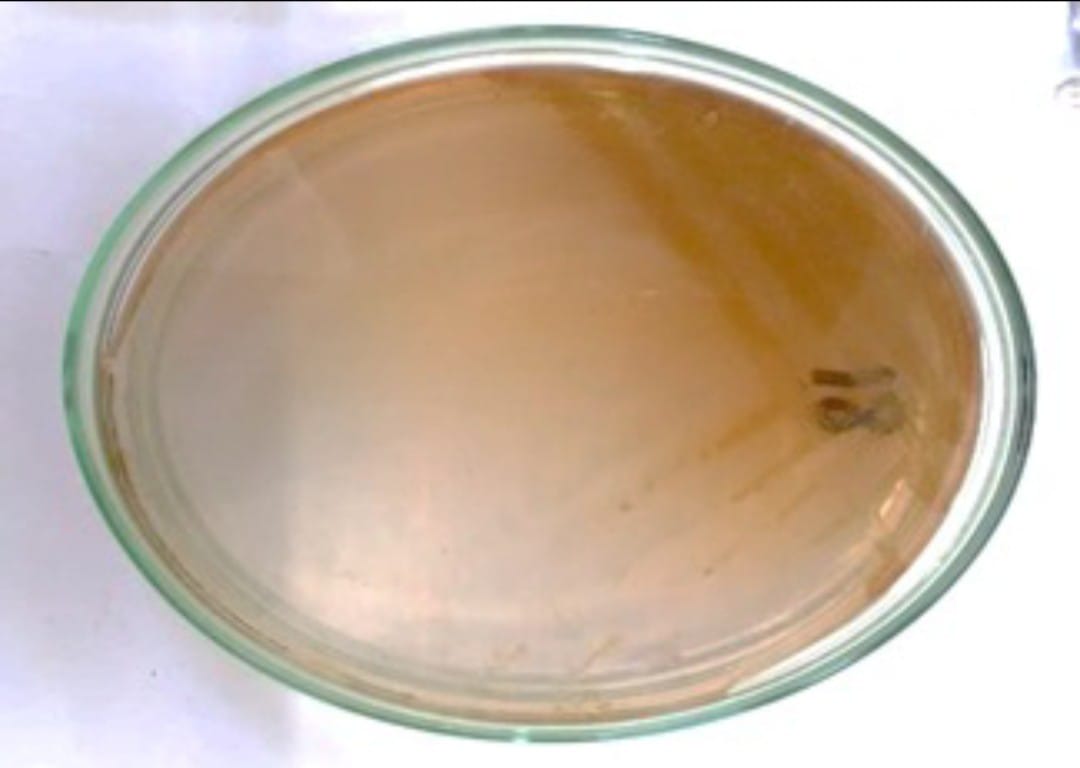


**A**

**D**

**C**

**B**

**Supplementary Figure S1.** **Morphological and biochemical characterization of** Stutzerimonas stutzeri **AUMC B-503.** (A) Gram-stained cells showing Gram-negative rod-shaped morphology. (B) Scanning electron micrograph (SEM) illustrating the bacillus-shaped cells at 3,500× magnification (scale bar = 5 µm). (C) Production of a melanin-like brown pigment on King’s B agar. (D) Positive catalase reaction evidenced by bubble formation upon exposure to hydrogen peroxide.


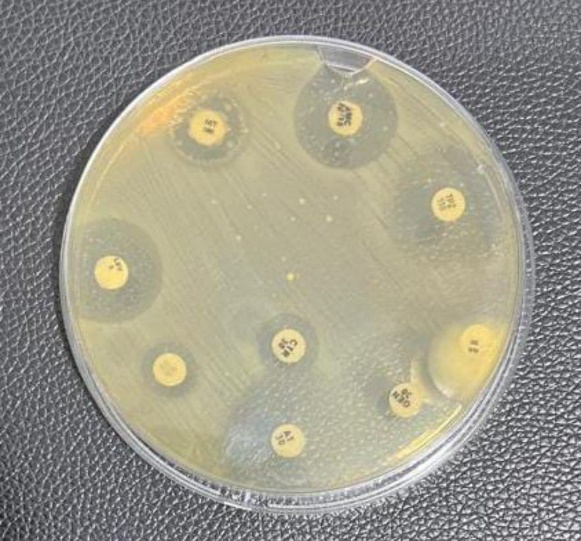

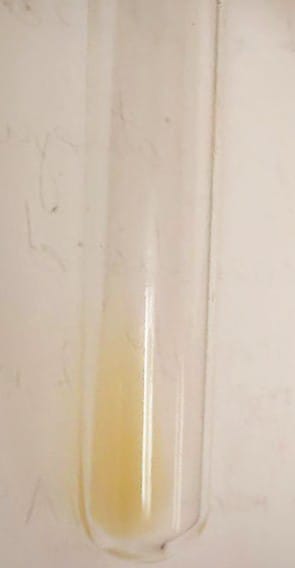

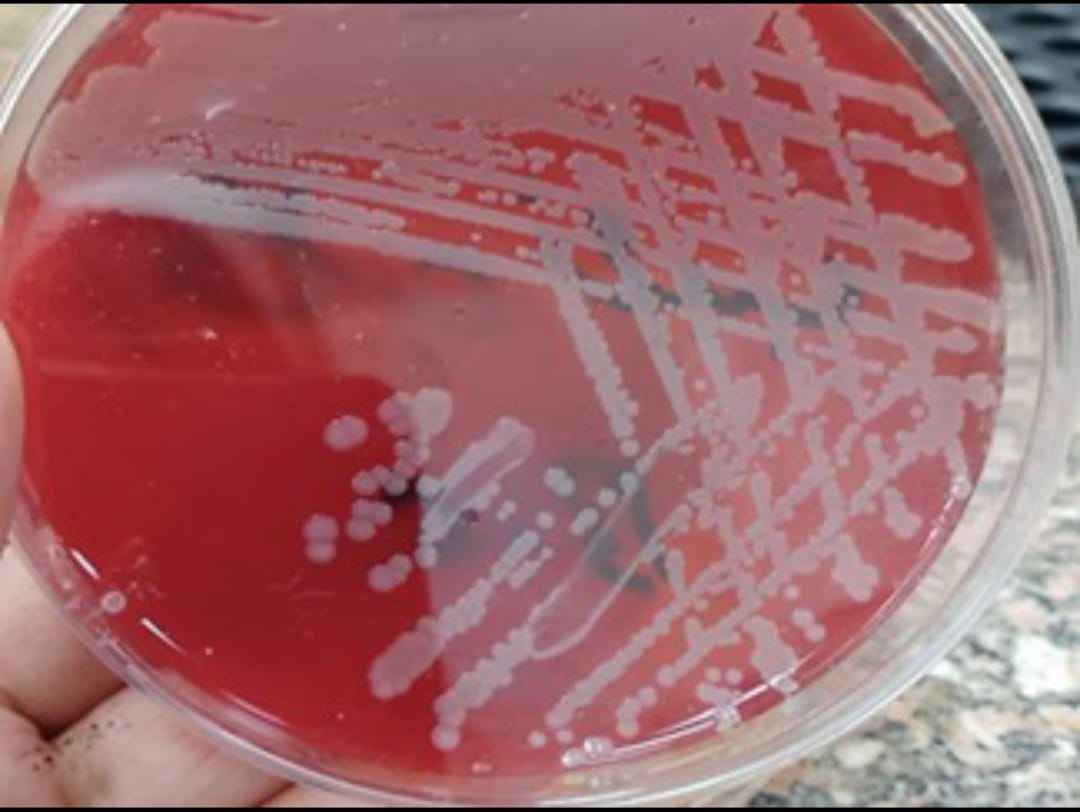


**A**

**B**

**C**

**Supplementary Figure S2.** **Biosafety assays of** Stutzerimonas stutzeri **AUMC B-503.** (A) Blood agar plate showing γ-hemolysis (non-hemolytic activity). (B) Negative coagulase reaction with absence of clot formation. (C) Antibiotic susceptibility test showing clear inhibition zones around all tested antibiotic discs, indicating sensitivity to all tested antibiotics.


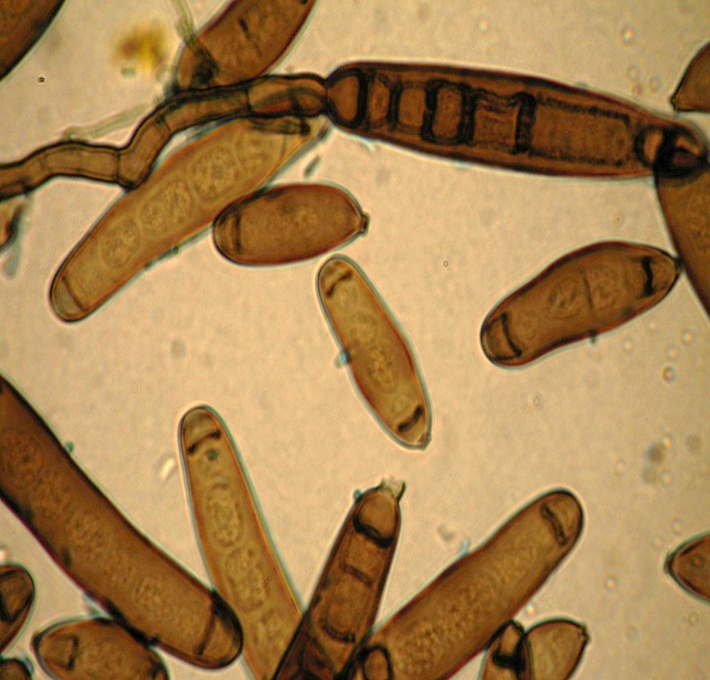

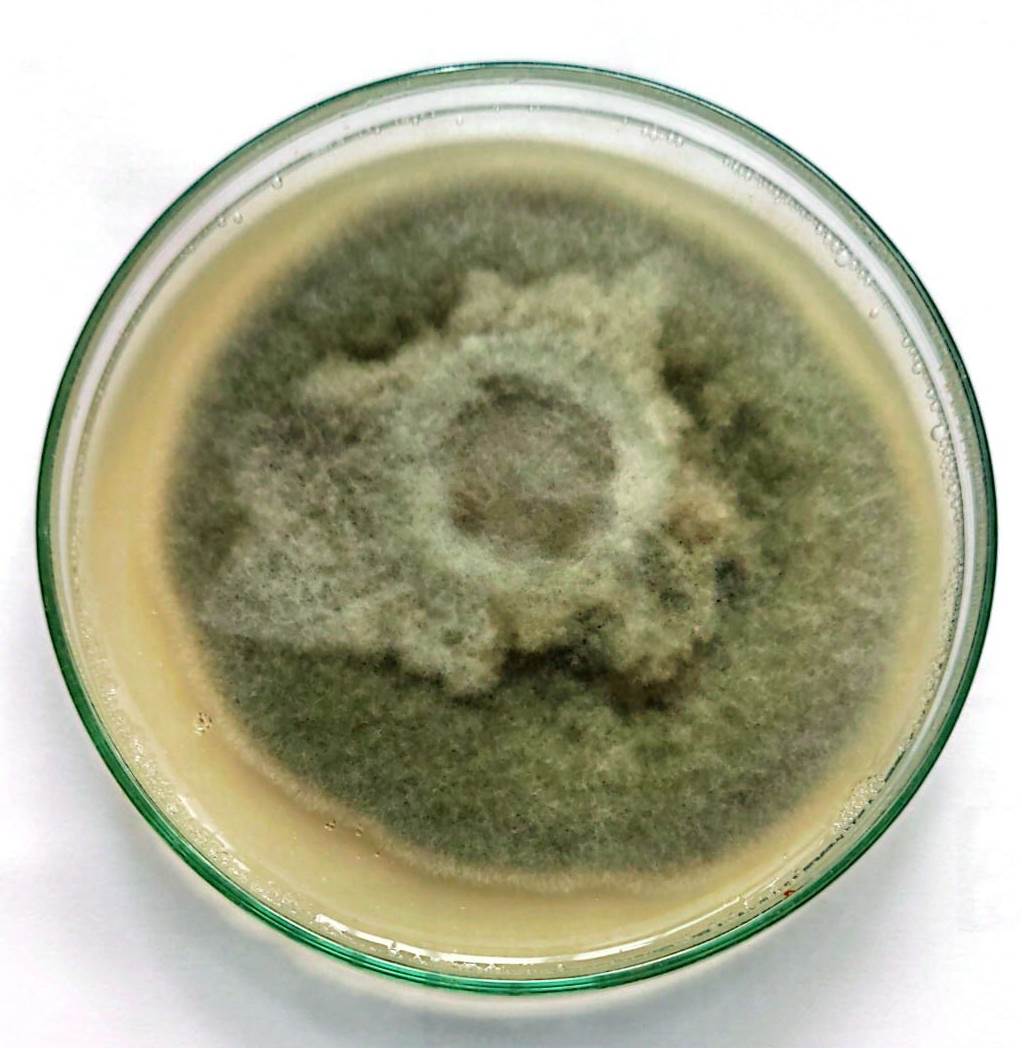


**A**

**B**

**Supplementary Figure S3.** Morphological characteristics of *Bipolaris oryzae* isolated from rice plants showing brown spot symptoms. (A) Colony morphology on potato dextrose agar (PDA) showing a grayish-olive, velvety surface with concentric ring pattern. (B) Microscopic view of dark, septate conidiophores producing brown, multiseptate, elliptical conidia (×1000 magnification).

**Supplementary Table 1.** Antibiotic classes, antimicrobial agents, disc potencies used and susceptibility pattern of selected strain (Stutzerimonas stutzeri **AUMC B-503).**

| **Antibiotic class** | **Antimicrobial agent** | **Disc potency (µg/Disc)** | **Diameter of inhibition zone (mm)** |
| --- | --- | --- | --- |
| Penicillin | Amoxicillin-Clavulanate (AMC) | 28/18 | 22 |
|  | Piperacillin/tazobactam (TPZ) | 110 | 30 |
| Cephalosporin | Ceftriaxone (CTR) | 30 | 14 |
| Aminoglycosides | Gentamicin (GEN) | 30 | 30 |
|  | Amikacin (AK) | 30 | 21 |
| Quinolones | Nalidixic acid (NA) | 30 | 16 |
|  | Levofloxacin (LEV) | 15 | 28 |
| Macrolide | Clindamycin (CD) | 20 | 12 |
| Monobactam | Aztreonam (AT) | 30 | 34 |

**Supplementary Table 2.** Primers used in this study.

| Gene | Abbreviation | Direction | Sequences (5^’^-3^’^) |
| --- | --- | --- | --- |
| Reference gene | ß-Actin | F | GGTAACATTGTGCTCAGTGGTGG |
|  |  | R | AACGACCTTAATCTTCATGCTGC |
| Chalcone synthase | *OsCHS* | F | CGGACTGGAACTCCATCTTC |
|  |  | R | TAAAAGATGACGTGTGGCGTA |
| Chalcone isomerase | *OsCHI* | F | TCCATCCTCTTCACCCACTC |
|  |  | R | TGTCAAACACGAGGGCAGTA |
| Flavonol synthase | *OsFLS* | F | ACTGCAAGCACATGCAGCGGC |
|  |  | R | CTTATGCAGGAACACCAGCTCGTCG |
| Ornithine aminotransferase | *OsOAT* | F | TGGCAAGTGCTGTGGCAGT |
|  |  | R | ACAGCTGCAGAAGCAGATC |
| Ethylene-responsive transcription Factor83 | *OsERF83* | F | CATGTCGCTTCAT CCTCACC |
|  |  | R | AGGTAGTCAGGTCCCAGGTC |
